# Activation of the hypoxia-inducible factor pathway protects against acute ischemic stroke by reprogramming central carbon metabolism

**DOI:** 10.7150/thno.88223

**Published:** 2024-04-29

**Authors:** Sarah Madai, Pinar Kilic, Rolf M. Schmidt, Carlos Bas-Orth, Thomas Korff, Michael Büttner, Glynis Klinke, Gernot Poschet, Hugo H. Marti, Reiner Kunze

**Affiliations:** 1 Institute of Physiology and Pathophysiology, Department of Cardiovascular Physiology, Heidelberg University, Heidelberg, Germany.; 2 Department of Medical Cell Biology, Institute for Anatomy and Cell Biology, Heidelberg University, Heidelberg, Germany.; 3 Metabolomics Core Technology Platform, Centre for Organismal Studies, Heidelberg University, Heidelberg, Germany.

**Keywords:** HIF, PHD, Roxadustat, ischemic stroke, metabolic reprogramming, aerobic glycolysis

## Abstract

Cell metabolism reprogramming to sustain energy production, while reducing oxygen and energy consuming processes is crucially important for the adaptation to hypoxia/ischemia. Adaptive metabolic rewiring is controlled by hypoxia-inducible factors (HIFs). Accumulating experimental evidence indicates that timely activation of HIF in brain-resident cells improves the outcome from acute ischemic stroke. However, the underlying molecular mechanisms are still incompletely understood. Thus, we investigated whether HIF-dependent metabolic reprogramming affects the vulnerability of brain-resident cells towards ischemic stress.

**Methods:** We used genetic and pharmacological approaches to activate HIF in the murine brain *in vivo* and in primary neurons and astrocytes *in vitro*. Numerous metabolomic approaches and molecular biological techniques were applied to elucidate potential HIF-dependent effects on the central carbon metabolism of brain cells. In animal and cell models of ischemic stroke, we analysed whether HIF-dependent metabolic reprogramming influences the susceptibility to ischemic injury.

**Results:** Neuron-specific gene ablation of prolyl-4-hydroxylase domain 2 (PHD2) protein, negatively regulating the protein stability of HIF-α in an oxygen dependent manner, reduced brain injury and functional impairment of mice after acute stroke in a HIF-dependent manner. Accordingly, PHD2 deficient neurons showed an improved tolerance towards ischemic stress *in vitro*, which was accompanied by enhanced HIF-1-mediated glycolytic lactate production through pyruvate dehydrogenase kinase-mediated inhibition of the pyruvate dehydrogenase. Systemic treatment of mice with roxadustat, a low-molecular weight pan-PHD inhibitor, not only increased the abundance of numerous metabolites of the central carbon and amino acid metabolism in murine brain, but also ameliorated cerebral tissue damage and sensorimotor dysfunction after acute ischemic stroke. In neurons and astrocytes roxadustat provoked a HIF-1-dependent glucose metabolism reprogramming including elevation of glucose uptake, glycogen synthesis, glycolytic capacity, lactate production and lactate release, which enhanced the ischemic tolerance of astrocytes, but not neurons. We found that strong activation of HIF-1 in neurons by non-selective inhibition of all PHD isoenzymes caused a HIF-1-dependent upregulation of 6-phosphofructo-2-kinase/fructose-2,6-bisphosphatase-3 redirecting glucose-6-phosphate from pentose phosphate pathway (PPP) to the glycolysis pathway. This was accompanied by a reduction of NADPH production in the PPP, which further decreased the low intrinsic antioxidant reserve of neurons, making them more susceptible to ischemic stress. Nonetheless, in organotypic hippocampal cultures with preserved neuronal-glial interactions roxadustat decreased the neuronal susceptibility to ischemic stress, which was largely prevented by restricting glycolytic energy production through lactate transport blockade.

**Conclusion:** Collectively, our results indicate that HIF-1-mediated metabolic reprogramming alleviates the intrinsic vulnerability of brain-resident cells to ischemic stress.

## Introduction

At the cellular level reduction of oxygen (O_2_)- and adenosine triphosphate (ATP)-consuming processes concomitant with maintenance of ATP generation are the most important adaptive reactions to hypoxic/ischemic stress. Among others the metabolic shift from O_2_-dependent mitochondrial oxidative phosphorylation (OXPHOS) towards the O_2_-independent glycolytic pathway, known as Pasteur effect, substantially contributes to the maintenance of sufficient ATP production under hypoxic/ischemic conditions 1, 2. Reprogramming of the glucose metabolism is primarily achieved by the hypoxia-inducible factor (HIF)-1-dependent upregulation of genes encoding glucose transporters and glycolytic enzymes 3-5, as well as the HIF-1 mediated suppression of OXPHOS by reducing metabolite entry into the tricarboxylic acid (TCA) cycle, activity of the electron transport chain (ETC) and mitochondrial mass 1, 2, 6, 7. Together, the increased glucose uptake and its subsequent glycolytic flux perpetuate sufficient cellular ATP production when O_2_ is scarce 1.

HIFs are mainly regulated by changes in protein stability and transcriptional activity in an O_2_-dependent manner. Under normoxic conditions, α subunits are hydroxylated on defined proline and asparagine residues by the family of prolyl-4-hydroxylase domain (PHD) proteins and factor inhibiting HIF (FIH), respectively, whose activity is dependent on O_2_, 2-oxoglutarate, ferrous iron and reducing compounds like ascorbate. While prolyl hydroxylation results in recruitment of the von Hippel-Lindau protein E3 ubiquitin ligase and immediate proteasomal degradation of HIF-1α and -2α, asparaginyl hydroxylation renders the HIF-α ​subunits unable to bind to their transcriptional co-​activators, the histone acetyl transferases CBP and p300, preventing the formation of a functional transcriptional complex 8, 9. Under hypoxic conditions, however, the enzymatic activity of the PHD and FIH HIF hydroxylases is largely reduced, thereby stabilizing the HIF-α subunits and increasing their transcriptional activity, respectively. The stabilized HIF-α subunits translocate into the nucleus, where they dimerize with HIF-1β and bind hypoxia response elements (HREs) within regulatory DNA regions of several hundred HIF-​responsive genes to enhance transcription 8, 9.

The adult human brain, accounting for a mere 2% of body weight, is estimated to be responsible for 20% of O_2_ consumption and 20-25% of glucose utilization 10. In normal conditions, the main source of energy for the brain is glucose that is utilized for the generation of energy in the form of ATP primarily from OXPHOS 10. Due to its high energy demand and its reliance on glucose and O_2_ for ATP generation, the brain is highly susceptible to hypoxic/ischemic injury.

We and others have already demonstrated that activation of the HIF signaling pathway in the rodent brain using genetic and pharmacological approaches alleviates tissue damage and functional impairments following ischemic stroke 11-18. The molecular mechanisms underlying the HIF-dependent neuroprotective effects in the context of acute ischemic stroke are, however, only insufficiently understood. Thus, in the present study we investigated the impact of both genetic and pharmacological activation of the HIF signaling pathway on the central carbon metabolism in the brain *in vivo* as well as in neurons and astrocytes *in vitro*. Using animal and cell models of ischemic stroke, we further analyzed whether potential HIF-dependent metabolic reprogramming affects the vulnerability towards ischemic stress.

## Methods

### Experimental animals

All transgenic mouse lines were established on a C57BL/6 background. We used female and male littermate mice that were age-matched between experimental groups. All animal experiments were approved by the local animal welfare committee (Regierungspräsidium Karlsruhe, Germany, permission number: 35-9185.81/G-45/18, 35-9185.81/G-74/23), conformed to the Guide for the Care and Use of Laboratory Animals published by the US National Institutes of Health and were performed in accordance with the recently published Animal Research: Reporting *In Vivo* Experiments (ARRIVE) guidelines (https://www.nc3rs.org.uk/arrive-guidelines). Mice were housed at constant room temperature (22 ± 2 °C) and relative humidity (50-55%) on a controlled 12:12 h light-dark cycle, and were provided with standard laboratory chow (LASQCdiet Rod16; LASvendi, Soest, Germany) and water *ad libitum*.

Mice that carried a floxed allele of *Phd2* (*Phd2 ^f/f^*) 19 alone or in combination with floxed alleles of *Hif1a* (*Hif1a ^f/f^*) 20 and *Hif2a* (*Hif2a ^f/f^*) 21 were crossed with *Camk2a:cre* mice 22 to conditionally knockout *Phd2*, *Hif1a* and *Hif2a* in forebrain neurons. Mice were genotyped using primers (Eurofins Genomics, Ebersberg, Germany) described in Table S1.

### Experimental stroke model

Mice were used at the age of 6-9 weeks. Mice were anesthetized by a mixture of 2% isoflurane, 70% N_2_O and remainder O_2_, and were maintained by reducing the isoflurane concentration to 1.0-1.5%. To induce focal cerebral ischemia a 7-0 silicon rubber-coated nylon monofilament (Doccol Corporation, Redlands, CA, USA) was introduced into the left internal carotid artery and pushed toward the left middle cerebral artery (MCA) as previously described 17. The intraluminal suture was left for 60 min. Subsequently, animals were re-anesthetized and the occluding monofilament was withdrawn to allow reperfusion for 3-24 h. For sham surgery, the mice underwent the same procedure without vessel occlusion. The animals were maintained at 37 °C during and after surgery until they were fully recovered from anesthesia. Then, mice were returned to their solitary cages in a heated (30 °C) environment with free access to food and water for 12 h. During the remaining time animals were kept under normal conditions as described above. Table S2 lists the criteria resulting in exclusion from end-point analysis.

Operators and investigators were blinded for mouse genotype or pharmacological treatment in all experiments and analyses. Evaluation of all read-out parameters was done independently and in a blinded fashion.

### Drug application *in vivo*

Roxadustat (FG-4592; Selleck Chemicals, Munich, Germany, #S1007) was dissolved in DMSO (10 mg/mL or 40 mg/mL) followed by dilution in 0.9% NaCl with 2% Tween-80 to a final concentration of 1 mg/mL or 4 mg/mL, respectively. Roxadustat was applied intraperitoneally (injection volume 0.2 mL per 20 g body weight) to 8-week-old male C57BL/6 mice at a dosage of either 10 mg/kg or 40 mg/kg body weight twice daily with 12 h between doses for 4 days. Control mice received equal volumes of vehicle solution (10% DMSO, 2% Tween-80 in 0.9% NaCl) twice a day for 4 days through intraperitoneal injection.

All mice were randomly allocated to experimental groups. Experimenters were blinded for animal treatment in all experiments and analyses. Evaluation of all read-out parameters was done independently and in a blinded fashion.

### Behavioral assessment

Neurological function was evaluated by using the Bederson neurological deficit score, according to the following scoring system: 0, no observable deficit; 1, forelimb flexion; 2, decreased resistance to lateral push; 3, unidirectional circling; 4, no movement 23. Alternatively, a modified neurological severity score was assessed to grade the neurologic function post-stroke on a scale of 0 to 14 (normal score 0; maximal deficit score 14) 24.

### Histopathological analyses

Animals were sacrificed by decapitation, brains were removed, and embedded into Tissue-Tek (Sakura Finetek, Staufen, Germany). From each brain, 24 coronal sections (10 µm thickness; 0.4 mm distance) were prepared using a Leica CM1520 cyrostat (Leica Biosystems, Wetzlar, Germany) at a constant temperature of -15 °C, and stained with cresyl violet (Merck Millipore, Darmstadt, Germany, #105235) according to manufacturer's instructions. Stained brain slices were digitized, and infarct and edema volume was measured using the image analysis software ImageJ (National Institutes of Health, Bethesda, MD, USA) as described previously 15, 25.

### Metabolome analysis by gas chromatography/mass spectrometry (GC/MS)

Animals were sacrificed by decapitation, brains were isolated, shock frozen in liquid nitrogen and stored at -80 °C until use. Frozen brain tissue was extracted with 190 µL 100% methanol containing 10 µL 0.02 mg/mL Ribitol for 15 min at 70 °C with vigorous shaking. After the addition of 100 µL 100% chloroform, samples were shaken at 37 °C for 5 min. To separate polar and organic phases, 200 µL HPLC-grade water were added and samples were centrifuged for 10 min at 11,000 x g. While avoiding the interphase containing cellular debris, 350 µL of the polar (upper) phase were transferred to a fresh glass vial and dried using a vacuum concentrator (Eppendorf Concentrator Plus) without heating. Sequential on-line methoximation and silylation reactions were performed using a MPS autosampler (Gerstel, Mülheim Ruhr, Germany). Methoximation was performed by adding 20 µL 20 mg/mL methoxyamine hydrochloride (Sigma-Aldrich, Steinheim, Germany, #226904) in pyridine (Sigma-Aldrich, #270970) and incubation at 37 °C for 90 min in a Gerstel MPS Agitator Unit (250 rpm). For silylation reactions, 45 µL of N-Methyl-N-(trimethylsilyl)trifluoroacetamide (MSTFA; Sigma-Aldrich, #69479) were added and samples were incubated at 37 °C for 30 min with gentle shaking. Before injection, samples were incubated at RT for 45 min. For GC/MS analysis, a GC-ToF system was used consisting of an Agilent 7890 Gas Chromatograph (Agilent Technologies, Waldbronn, Germany) fitted with a Rxi-5Sil MS column (30 m x 0.25 mm x 0.25 µm; Restek GmbH, Bad Homburg, Germany) and coupled to a Pegasus BT Mass Spectrometer (LECO, Mönchengladbach, Germany). The GC was operated with an injection temperature of 250 °C and 1 µL sample was injected in splitless mode. The GC temperature program started with a 1 min hold at 40 °C followed by a 6 °C/min ramp up to 210 °C, a 20 °C/min ramp up to 330 °C and a bake-out at 330 °C for 5 min using Helium as carrier gas with constant linear velocity. The ToF mass spectrometer was operated with ion source and interface temperatures of 250 °C, a solvent cut time of 9 min and a scan range (m/z) of 50 - 600 with an acquisition rate of 17 spectra/s. The ChromaTof v5.50 software (LECO) was used for data processing.

Metabolite Set Enrichment Analysis (MSEA) was performed using MetaboAnalyst 6.0 software (http://www.metaboanalyst.ca) 26 with the following settings: module: enrichment analysis; type of enrichment analysis: quantitative enrichment analysis; compound ID: compound names; missing value estimation: Bayesian principal component analysis (BPCA); data filtering: none; sample normalization: none; sample transformation: none; data scaling: none; metabolite set library: pathway-based (KEGG; 80 metabolite sets based on KEGG human metabolic pathways). Pathways with raw *p* values < 0.05 were considered as statistically significant.

### Laser speckle contrast imaging (LSCI)

Cortical blood flow (CBF) was monitored using a two-dimensional laser speckle contrast analysis system (PeriCam PSI High Resolution with PIMSoft; Perimed, Stockholm, Sweden). Mice were anesthetized with isofluorane and maintained at physiological body temperature as described above. A midline incision was made in the scalp and the skull surface cleaned with sterile normal saline. A charged-coupled device camera was placed 9.5 cm above the skull using a Pericam PSI System and blood perfusion images were taken at 30 min after MCAO and at 3 h upon onset of reperfusion. Raw speckle images were taken in a 1.8 cm × 1.6 cm field (at 1 frame/s). Ten frames averaging, with the resolution of 0.02 mm six consecutive images at each time point per animal were averaged for analysis of CBF expressed in arbitrary units (perfusion units). The entire ipsilateral and contralateral hemispheres were defined as the main regions of interest (ROIs). Across the ipsilateral hemisphere further ROIs were determined at 30 min post-MCAO based on the following blood flow thresholds: infarct core (CBF < 40% of mean contralateral hemisphere), hypoperfused peri-infarct region (CBF between 40 and 70% of mean contralateral hemisphere).

### Organotypic hippocampal slice cultures (OHSC)

Mice (P6-8) were decapitated, brains were aseptically removed, and hippocampi were dissected under sterile conditions in ice-cold isolation buffer (pH 7.2) containing Gey's balanced salt solution (Thermo Fisher Scientific, Dreieich, Germany, #J67569.K2), 1 mM kynurenic acid (a NMDA receptor antagonist; Sigma-Aldrich, #K3375) and 0.5% glucose. Hippocampi were cut into 400 μm slices using a McIlwain tissue chopper. Slices were placed onto Millicell-CM membranes (Merck Millipore, #PICM03050; three slices on each membrane). Membranes were transferred to a six-well dish with 1 mL ice-cold isolation buffer, and were incubated for 30-60 min at 4 °C. Subsequently, the isolation buffer was replaced with Eagle's basal medium (Thermo Fisher Scientific, #41010026) including 25% Hank's balanced salt solution (Thermo Fisher Scientific, #24020091), 25% horse serum (Thermo Fisher Scientific, #16050122), 1 mM L-glutamine (Thermo Fisher Scientific, #25030024) and 0.5% glucose, and OHSCs were maintained at interface between air and medium in a humidified incubator with 5% CO_2_ in air and 37 °C for 10 days.

### Primary cell cultures

Cortical neurons from neonatal mice (P0-1) were prepared and maintained as described previously 27. Experiments were performed after a culturing period of 10 days *in vitro* (DIV) when neurons express functional glutamate receptors and have developed an extensive network of synaptic contacts. At 8 DIV, growth medium was replaced with defined serum-free culture medium consisting of a mixture of a buffered saline solution (10 mM HEPES, pH 7.4, 114 mM NaCl, 26.1 mM NaHCO_3_, 5.3 mM KCl, 1 mM MgCl_2_, 2 mM CaCl_2_, 5 mM glucose, 1 mM glycine, 0.5 mM C_3_H_3_NaO_3_, and 0.001% phenol red) and phosphate-free Eagle's minimum essential medium (9:1 v/v) supplemented with insulin (7.5 μg/mL), transferrin (7.5 μg/mL), and sodium selenite (7.5 ng/mL) (ITS supplement, Merck Millipore, #I3146).

Primary mixed glial cell cultures were prepared from the brains of newborn mice (P0-2) as previously described 28. After 5 days, residual microglial cells were eliminated by treatment with 50 mM L-leucine methyl ester (Merck Millipore, #L1002) for 1 h, and microglia-free astrocytes were cultured for a further 7-9 days.

### Oxygen-glucose deprivation (OGD)

Cells were treated with roxadustat (Selleck Chemicals) in the presence or absence of either 5 mM sodium dichloroacetate (DCA; Merck Millipore, #347795) or 10 nM AZ-PFKFB3-67 (Tocris-Bio-Techne, Wiesbaden, Germany, #5742) for 24 h. Subsequently, cells were maintained for 4 h in artificial cerebral spinal fluid (aCSF, pH 7.4) containing 116 mM NaCl, 5,4 mM KCl, 0,8 mM MgSO_4_, 1 mM Na_2_HPO_4_, 26,2 mM NaHCO_3_, 1 mM Glycin, and 1,8 mM CaCl_2_ in an Invivo2 Plus hypoxia workstation (Ruskinn, Leeds, UK) flooded with humidified gas mixture consisting of 5% CO_2_ and ~95% N_2_ (0.2% O_2_).

OHSCs were treated with roxadustat (Selleck Chemicals) in the presence or absence of 10 µM syrosingopine (Sigma-Aldrich, #SML1908) for 24 h following 30 min exposure to 0.2% O_2_ in aCSF (pH 7.6) containing 125 mM NaCl, 2.5 mM KCl, 2 mM MgSO_4_, 1.25 mM Na_2_HPO_4_, 25 mM NaHCO_3_, 2 mM CaCl_2_, and 10 mM sucrose. Then, OHSCs were returned to normoxic conditions, aCSF was replaced by normal growth medium, and slices were cultured for further 24 h simulating post-ischemic reperfusion *in vitro*.

### Quantification of cell death

Cell death in primary cell cultures was either quantified using the CytoTox-Glo Cytotoxicity Assay (Promega, Walldorf, Germany, #G9290) or the LDH Cytotoxicity Assay Kit (Thermo Fisher Scientific, #88953) according to manufacturer's specifications.

Propidium iodide (5 µg/mL, Sigma-Aldrich, #P4864) was added to OHSCs and incubated for 30 min. Following fixation with 4% paraformaldehyde for 30-60 min and nuclear staining with 0.02% DAPI (Sigma-Aldrich, #D9542) for 10 min, slices were scanned in a 20-fold magnification by using a Zeiss Axiovert 200M microscope equipped with Hamamatsu Orca Flash 4.0 camera and TissueFAXS scanning software (TissueGnostics, Vienna, Austria). Cell death was quantified in three size-matched hippocampal CA sub regions per sample. Total cumulated nucleus-associated DAPI and PI fluorescence was assessed in a blinded fashion by apply an automated detection method (cellSens Dimension 1.18, Olympus, Count and Measure tool). The level of cell death was calculated as PI/DAPI fluorescence ratio.

### Real-time cell metabolic analysis

Oxygen consumption rate (OCR), extracellular acidification rate (ECAR) and proton efflux rate (PER) were determined using the Seahorse XFe96 Analyzer (Agilent). Cell glycolytic profile was evaluated by using the Glycolytic Rate Assay (Agilent, #103344-100) according to manufacturer's instructions. Briefly, cells were plated in Seahorse XFe96 cell culture microplates (Agilent, #103794-100), and were cultured and treated with 10 µM roxadustat for 24 h as described above. Prior to the assay, cells were washed and incubated for 1 h with XF DMEM medium supplemented with 5 mM glucose, 1 mM pyruvate and 2 mM L-glutamine (Agilent, #103680-100), and in atmospheric air at 37 °C. Seahorse XFe96 cartridges (Agilent, #103793-100) were hydrated according to the manufacturer's instructions. PER and OCR were measured under basal conditions and in response to 0.5 mM rotenone/antimycin A (Rot/AA; inhibitors of mitochondrial electron transport chain) and 50 mM 2-deoxy-D-glucose (2-DG; glycolysis inhibitor). Glycolytic PER (GlycoPER) was calculated as described elsewhere 29 to determine basal glycolysis and compensatory glycolysis (in response to Rot/AA). Cell Mito Stress Test was applied to estimate key parameters of mitochondrial respiration. OCR and ECAR were measured under basal conditions and in response to sequential injections of 1 µg/mL oligomycin (ATP Synthase / Complex V inhibitor; Merck Millipore, #495455), 2 µM CCCP (carbonylcyanide-3-chlorophenylhydrazone, mitochondrial uncoupler; Merck Millipore, #C2759), and 1 µM rotenone (Merck Millipore, #R8875) plus 1 mg/mL antimycin A (Merck Millipore, #A8674) to calculate basal respiration (= OCR_basal_ - OCR_Rot/AA_), ATP production-linked respiration (= (OCR_basal_ - OCR_Oligo_) - OCR_Rot/AA_) and maximal respiration (= OCR_CCCP_ - OCR_Rot/AA_).

### Measurements of glucose consumption and lactate production

L-lactate concentration was determined according to a previously published protocol 30. In brief, samples in 96-well microtiter plates were mixed with reaction buffer (0.1 M citric acid, 10 mg/mL BSA, 0.1% CaCl_2_; pH 6.0) containing 0.25 units/mL lactate oxidase (Sigma-Aldrich, #L9795), 0.25 units/mL horseradish peroxidase (Sigma-Aldrich, #P8375) and 500 µM ABTS (Merck Millipore, #11204521001). After incubation for 1 h at room temperature, absorbance at 405 nm was determined.

Alternatively, lactate concentration in cell culture supernatants or mouse brain tissue homogenates was determined using the Colorimetric L-lactate assay kit (Merck Millipore, #MAK329) according to the supplier's protocol.

Glucose concentration in cell culture supernatants was determined according to a previously published protocol 31. In brief, samples in 96-well microtiter plates were mixed with reaction buffer (177 mM Tris HCl, 10 mM MgCl_2_; pH 7.8) containing 5 mM ATP (Merck Millipore, #10127523001), 2.5 mM nicotinamide adenine dinucleotide phosphate (NADP; Carl Roth, Karlsruhe, Germany, #AE13.1), 3 U/mL hexokinase (Merck Millipore, #11426362001), 3.4 U/mL glucose-6-phosphate dehydrogenase (Merck Millipore, #10165875001). After incubation for 1 h at room temperature, absorbance at 340 nm was determined.

### Glycogen quantification

Cellular glycogen content was measured fluorometrically using the Glycogen Colorimetric/Fluorometric Assay Kit (Abcam, Cambridge, UK, #ab65620) according to manufacturer's instructions.

### Detection of thiol compounds

Thiols were extracted from 1x10^6^ cells with 0.3 mL of 0.1 M HCl in an ultrasonic ice-bath for 10 min. The resulting homogenates were centrifuged twice for 10 min at 4 °C and 16.400 x g to remove cell debris. Total glutathione was quantified by reducing disulfides with dithiothreitol (DTT) followed by thiol derivatization with the fluorescent dye monobromobimane (Thiolyte; Merck Millipore, #596105). For quantification of GSSG, free thiols were first blocked by N-ethylmaleimide followed by DTT reduction and monobromobimane derivatization. GSH equivalents were calculated by subtracting GSSG from total glutathione levels. Derivatization was performed as described previously 32. Separation was carried out using Acquity H-class UPLC-FLR system (Waters, Eschborn, Germany) with a binary gradient of buffer A (100 mM potassium acetate, pH 5.3) and solvent B (acetonitrile) with the following gradient: 0 min 2.3% buffer B; 0.99 min 2.3%, 1 min 70%, 1.45 min 70%, and re-equilibration to 2.3% B in 1.05 min at a flow rate of 0.85 mL min^-1^. The column (Acquity BEH Shield RP18 column, 50 mm x 2.1 mm, 1.7 µm, Waters) was maintained at 45 °C and sample temperature was kept constant at 14 °C. Monobromobimane conjugates were detected by fluorescence at 480 nm after excitation at 380 nm after separation and quantified using ultrapure standards (Sigma-Aldrich). Data acquisition and processing was performed with the Empower3 software suite (Waters).

### Detection of energy-carrier metabolites

Frozen cell pellets (1x10^6^ cells) were processed following an adjusted protocol targeting energy carriers such as nicotinamide adenine dinucleotide (NAD)/NADH and NADP/NADPH 33. We extended this liquid chromatography (LC)-MS/MS method by including polarity switching and additional metabolites of interest. Briefly, cell pellets were extracted on ice using 250 µL cooled extraction buffer (Acetonitrile : MeOH : 15 mM ammonium acetate in H_2_O (3:1:1), pH 10) . Subsequently, samples were sonicated to ensure complete disruption of all cells using a sonication bath (Transsonic 460, Elma Sonic, Singen, Germany) for 5 min at the highest frequency on ice. Afterwards, samples were centrifuged for 15 min at 4 °C and 13,000 x g, and the resulting supernatant was transferred to a new LC-MS grade autosampler vial and immediately frozen at -80 °C if instrument was not directly available for measuring.

For metabolite separation and detection, an Acquity I-class Plus UPLC system (Waters) coupled to a QTRAP 6500+ (AB Sciex, Framingham, MA, USA) mass spectrometer with electrospray ionization (ESI) source was used. In detail, metabolites were separated on an Acquity Premier BEH Amide Vanguard Fit column (100 mm × 2.1 mm, 1.7 µm, Waters) with constant column temperature of 35 °C. Separation of NAD/NADH, NADP/NADPH and additional energy carriers was achieved by the following LC gradient scheme (Table S3) using mobile phase A (5 mM ammonium acetate in H_2_O + 0.05% (v/v) ammonium hydroxide, pH 10) and mobile phase B (Acetonitrile + 0.05% (v/v) ammonium hydroxide, pH 10). An overview of multiple-reaction monitoring transitions, retention times, and MS parameters is provided in Table S4. Data acquisition was performed using Analyst 1.7.2 (AB Sciex) and processed using the OS software suite 2.0.0 (AB Sciex).

### Immunoblotting

Isolation of total, cytosolic and nuclear protein fractions from brain tissue and cells, sodium dodecyl sulfate polyacrylamide gel electrophoresis and immunoblotting were conducted as reported previously 34, 35. Antibodies used are given in Table S5.

### Quantitative real-time RT-PCR analysis

RNA isolation from tissue samples or cells, cDNA synthesis, and quantitative real-time PCR were performed as described recently 36. Primers were purchased from Eurofins Genomics (for primer sequences, see Table S6).

### Statistical analysis

Data plotting and statistical analyses were done in Prism 8 (GraphPad Software, La Jolla, CA, USA). If not indicated otherwise, all results are expressed as mean ± standard deviation (SD). Differences between two independent experimental groups were analyzed by two-tailed Student's *t* test (numerical data) or Mann-Whitney *U* rank-sum test (ordinal data). Differences in one parameter between three or more independent experimental groups were analyzed by using one-way ANOVA followed by a Holm-Sidak's multiple comparisons test. Differences in two parameters between two or more independent experimental groups were analyzed by two-way ANOVA followed by a Holm-Sidak's multiple comparisons test. A probability value of *P* < 0.05 was considered statistically significant.

## Results

### Neuronal PHD2 deficiency increases HIF-α protein levels in the postnatal murine brain

Neuron-specific gene knock-out mice were generated by crossing animals harboring two floxed alleles of the targeted gene(s) with transgenic mice expressing Cre recombinase under control of the *calcium/calmodulin-dependent protein kinase II alpha* (*Camk2a*) gene promoter. We and others provided experimental evidence that *Camk2a* gene promotor-driven Cre/loxP recombination is restricted to post-mitotic forebrain neurons, and starts just before birth 17, 22, 37.

**Figure 1:**
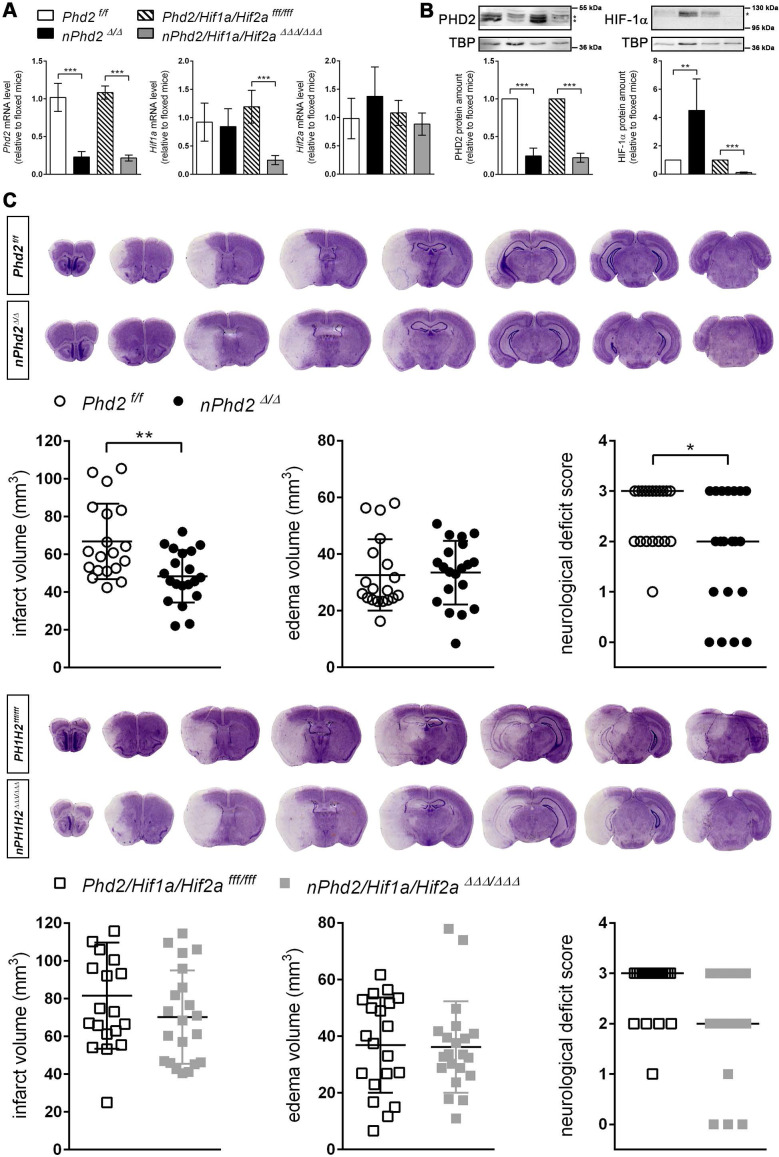
** Neuron-specific PHD2 inactivation reduces brain injury and functional impairment after acute stroke through HIF-dependent mechanisms. (A)** RNA and **(B)** nuclear proteins were prepared from forebrain of adult *Phd2 ^f/f^*, *nPhd2 ^Δ/Δ^*, *Phd2/Hif1a/Hif2a ^fff/fff^* and *nPhd2/Hif1a/Hif2a ^ΔΔΔ/ΔΔΔ^* mice. **(A)** *Phd2*, *Hif1a* and *Hif2a* transcript levels were determined by quantitative real-time RT-PCR. Values are normalized to *Rps12* and expressed as fold change to *Phd2 ^f/f^* or *Phd2/Hif1a/Hif2a ^fff/fff^*, respectively (*n* = 4-5 per group; unpaired two-tailed Student's *t* test; *** *p* < 0.001). **(B)** Nuclear PHD2 and HIF-α protein levels were quantified by Western blotting (protein bands used for densitometric analysis are marked with an asterisk). Values are normalized to TBP and expressed as fold change to *Phd2 ^f/f^* or *Phd2/Hif1a/Hif2a ^fff/fff^*, respectively (*n* = 4-5 per group; unpaired two-tailed Student's *t* test; ** *p* < 0.01, *** *p* < 0.001). **(C)** Mice underwent 60 min of MCAO followed by 24 h reperfusion. Infarct and edema volume were determined by cresyl violet staining (*n* = 19-21 per group; unpaired two-tailed Student's *t* test; * *p* < 0.05, ** *p* < 0.01). Neurological function was assessed using the Bederson neurological deficit score (median; *n* = 19-21 per group; Mann-Whitney *U* rank-sum test; * *p* < 0.05).

Neuron-specific *Phd2* gene ablation reduced total *Phd2* mRNA and PHD2 protein levels in the forebrain of adult mice by about 80% (Figure 1). Genetic inactivation of both *Hif1a* and *Hif2a* restricted to neurons resulted in a significant reduction of *Hif1a* mRNA (-80%) and HIF-1α protein (-90%), whereas *Hif2a* mRNA levels were only slightly decreased (-18%) in the murine forebrain (Figure 1A-B). As compared to *Phd2 ^f/f^* littermates, HIF-1α protein but not *Hif1a* mRNA levels were significantly increased in *Phd2* deficient animals (Figure 1A-B). These findings are in accordance with available single-cell RNA-seq datasets identifying neurons as major source for *Phd2* and *Hif1a* transcripts among all cell types of the adult murine brain, whereas neuronal *Hif2a* mRNA levels are the lowest among all brain-resident cells (Figure S1).

### Neuron-specific PHD2 inactivation reduces brain injury and functional impairment after acute stroke through HIF-dependent mechanisms

The infarct lesion size was significantly reduced in neuron-specific *Phd2* deficient (*nPhd2 ^Δ/Δ^*) mice, while brain swelling did not differ markedly between* nPhd2 ^Δ/Δ^* and *Phd2 ^f/f^* mice subjected to acute ischemic stroke (Figure 1C). Decreased brain tissue injury due to loss of neuronal PHD2 was found in both sexes (♀: 66 ± 7 vs 50 ± 3 mm^3^, *p* = 0.044, *n* = 9; ♂: 68 ± 7 vs 47 ± 5 mm^3^, *p* = 0.024, *n* = 10-11). In accordance to our histopathological results, functional neurological impairment was significantly less pronounced in *nPhd2 ^Δ/Δ^* mice as compared to *Phd2 ^f/f^* animals suffering from acute ischemic stroke (Figure 1C). Weight loss and mortality of *nPhd2 ^Δ/Δ^* mice due to ischemia/reperfusion (I/R) injury were comparable to *Phd2 ^f/f^* littermates (Figure S2). Previously, we demonstrated that the improved health status of *nPhd2 ^Δ/Δ^* mice after acute stroke is accompanied by activation of the HIF signaling pathway 17. To address whether acute brain protection in *nPhd2 ^Δ/Δ^* mice requires activation of the HIF pathway, mice with triple neuronal inactivation of PHD2, HIF-1α and HIF-2α (*nPhd2/Hif1a/Hif2a ^ΔΔΔ/ΔΔΔ^*; Figure 1A-B) were generated, and subjected to cerebral I/R. In the acute stage upon focal ischemic stroke neuroprotection as seen in *nPhd2 ^Δ/Δ^* mice was absent, as infarct lesion size (♀: 88 ± 25 vs 71 ± 5 mm^3^, *p* = 0.122, *n* = 10-12; ♂: 75 ± 30 vs 69 ± 26 mm^3^, *p* = 0.680, *n* = 9-10), cerebral edema and neurological impairment were comparable in* nPhd2/Hif1a/Hif2a ^ΔΔΔ/ΔΔΔ^* and *Phd2/Hif1a/Hif2a ^fff/fff^* mice (Figure 1C). Post-stroke weight loss and mortality were not significantly different between both genotypes (Figure S2). Conclusively, our data indicate that improved central nervous system (CNS) preservation and neurological dysfunction after acute stroke in *Phd2* deficient mice are dependent on the presence of HIF-α in neurons.

### Improved ischemic tolerance of PHD2 deficient neurons is accompanied by augmented glucose uptake and glycolytic flux as well as glucose metabolism switch from oxidative phosphorylation to aerobic glycolysis in a HIF-dependent manner

**Figure 2:**
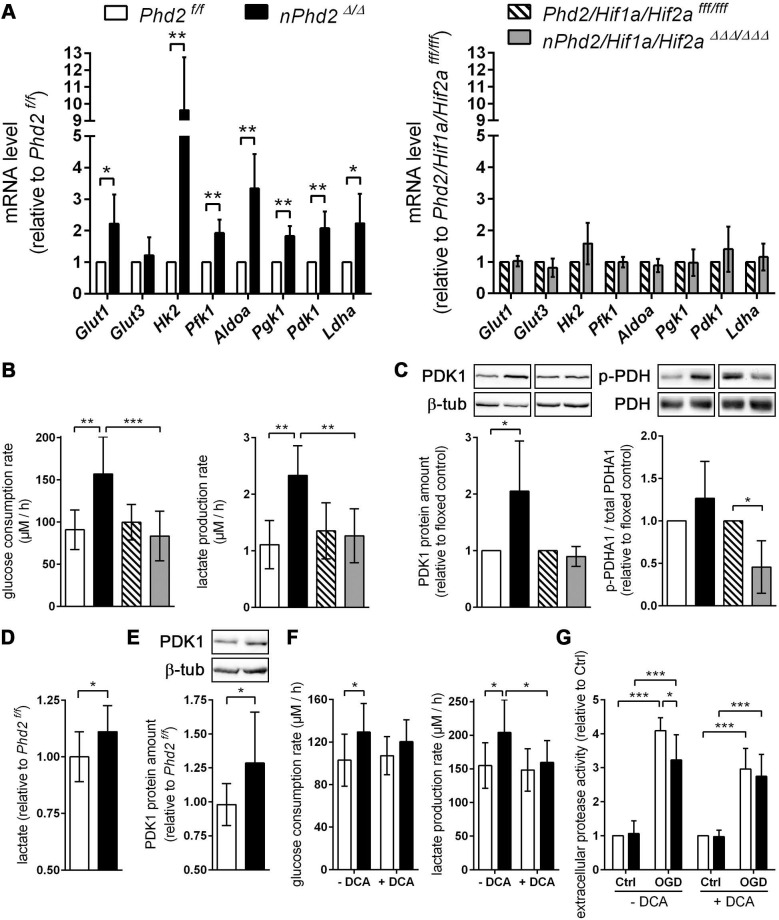
** PHD2 deficiency in neurons promotes a HIF-dependent metabolic switch from mitochondrial oxidative phosphorylation to aerobic glycolysis. (A)** Real-time RT-PCR was used to determine transcript levels of HIF target genes involved in glucose metabolism in neurons derived from neonatal *Phd2 ^f/f^*, *nPhd2 ^Δ/Δ^*, *Phd2/Hif1a/Hif2a ^fff/fff^* and *nPhd2/Hif1a/Hif2a ^ΔΔΔ/ΔΔΔ^* mice. Values are normalized to *Rps12* and expressed as fold change to *Phd2 ^f/f^* or *Phd2/Hif1a/Hif2a ^fff/fff^*, respectively (*n* = 4-5 per group; unpaired two-tailed Student's *t* test; * *p* < 0.05, ** *p* < 0.01). **(B)** Glucose consumption and extracellular lactate levels were measured in neurons cultured for 24 h under low glucose conditions (*n* = 4-11 per group; One-way ANOVA with Holm-Sidak's multiple comparisons test; ** *p* < 0.01, *** *p* < 0.001). **(C)** PDK1, p-PDHA1 and total PDHA1 protein levels were quantified by Western blotting. Values are normalized to β-tubulin and expressed as fold change to *Phd2 ^f/f^* or *Phd2/Hif1a/Hif2a ^fff/fff^*, respectively (*n* = 5-11 per group; unpaired two-tailed Student's *t* test; * *p* < 0.05). **(D)** Lactate was quantified in the forebrain of adult *Phd2 ^f/f^* and *nPhd2 ^Δ/Δ^* mice. Values are expressed as fold change to *Phd2 ^f/f^* (*n* = 13 per group; unpaired two-tailed Student's *t* test; * *p* < 0.05).** (E)** PDK1 protein level in the forebrain was quantified by Western blotting. Values are normalized to β-tubulin and expressed as fold change to *Phd2 ^f/f^* (*n* = 10 per group; unpaired two-tailed Student's *t* test; * *p* < 0.05).** (F)** Glucose consumption and extracellular lactate levels were measured in neurons cultured for 24 h in the presence or absence of 5 mM dichloroacetate (DCA) (*n* = 8-12 per group; Two-way ANOVA with Holm-Sidak's multiple comparisons test; * p < 0.05). **(G)** Neurons were cultured in the absence or presence of 5 mM DCA for 24 h prior to exposure to OGD conditions (glucose-free aCSF; 0.2% O_2_) for 4 h. Cells exposed to aCSF (+Glc) under normoxic conditions for 4 h are used as Ctrl. Cell viability was determined using a dead-cell protease activity assay. Values are expressed as fold change of *Phd2 ^f/f^* cells exposed to normoxic conditions (*n* = 5 per group; Two-way ANOVA with Holm-Sidak's multiple comparisons test; * *p* < 0.05, *** *p* < 0.001).

In order to elucidate whether potential HIF-dependent reprogramming of the neuronal metabolism contributes to the improved outcome after ischemic stroke, we first analyzed the expression of HIF target genes, whose function is related to glucose metabolism, in neuronal cultures derived from newborn *nPhd2 ^Δ/Δ^* and* nPhd2/Hif1a/Hif2a ^ΔΔΔ/ΔΔΔ^* mice. Neurons prepared from *nPhd2 ^Δ/Δ^* mice showed a significant reduction of *Phd2* mRNA (-60%), whereas *Hif1a* transcript levels remained unchanged (Figure S3). In neuronal cultures originated from* nPhd2/Hif1a/Hif2a ^ΔΔΔ/ΔΔΔ^* mice *Phd2* and *Hif1a* mRNA levels were significantly decreased by about 65% (Figure S3).* Phd2* deficient neurons exhibited significantly increased mRNA levels of glucose transporter 1 (*Glut1*) and glycolytic enzymes including hexokinase 2 (*Hk2*), phosphofructokinase 1 (*Pfk1*), fructose-bisphosphate aldolase A (*Aldoa*), phosphoglycerate kinase 1 (*Pgk1*), and lactate dehydrogenase A (*Ldha*) (Figure 2A).

Additional genetic ablation of *Hif1a* and *Hif2a* completely prevented the transcriptional up-regulation of all metabolic enzymes (Figure 2A). Accordingly, glucose uptake and extracellular lactate levels were significantly increased in *Phd2* deficient neurons, whereas it remained unchanged in *Phd2/Hif1a/Hif2a* deficient neurons (Figure 2B). Additionally, HIF-1 signaling is considered to minimize reactive oxygen species (ROS) production from the mitochondria during hypoxic/ischemic conditions by reducing the mitochondrial mass, and also through regulating the expression of alternative isoforms of subunits of the respiratory complexes 2. HIF-1 has been shown to upregulate an isoform of the complex IV subunit COX4, namely cytochrome c oxidase subunit 4 isoform 2 (COX4I2), which makes electron transfer and O_2_ consumption more efficient in hypoxia 2. Moreover, HIF-1 is believed to upregulate the mitochondrial lon protease 1 (LonP1), which is required for the degradation of the less efficient COX4I1 subunit 2. In *Phd2* deficient neurons the transcript levels of *Cox4i2* were significantly increased, whereas *Lonp1* expression was not affected (Figure S3). HIFs have also been shown to positively regulate the expression of the transcriptional repressor, deleted in esophageal cancer 1 (DEC1), which suppresses peroxisome proliferator-activated receptor gamma coactivator 1 alpha (PGC-1α) expression, and thus leads to decreased mitochondrial biogenesis 2. Concordantly, in *Phd2* deficient neurons the expression of *Dec1* was significantly increased, whereas the expression of *Ppargc1a* was significantly decreased (Figure S3). In addition to the suppression of mitochondrial biogenesis, it has been reported that HIFs promote mitochondrial turnover by up-regulation of BCL2/adenovirus E1B 19 kDa protein-interacting protein 3 (BNIP3) and BNIP3-like (BNIP3L/NIX), which flag mitochondria for degradation by the autophagy pathway 2. At mRNA level, both *Bnip3* and *Bnip3l* were significantly upregulated in *Phd2* deficient neurons (Figure S3). The altered expression of genes regulating mitochondrial mass and ROS production in *Phd2* deficient neurons was completely reversed by additional genetic ablation of *Hif1a* and *Hif2a* (Figure S3).

Furthermore, mRNA and protein levels of the HIF-1 targeted gene pyruvate dehydrogenase kinase 1 (*Pdk1*) were significantly increased in *Phd2* deficient neurons, but not in* Phd2/Hif1a/Hif2a* deficient nerve cells (Figure 2A and 2C). PDK1 inactivates the pyruvate dehydrogenase (PDH) complex through inhibitory serine phosphorylation of the pyruvate dehydrogenase E1 alpha subunit (PDHA1), preventing pyruvate from being converted to acetyl Co-A, thus hindering the initiation of the TCA cycle 1, 6, 7. Instead, cytosolic LDH catalyzes the conversion of pyruvate to lactate and concurrently generates NAD^+^, a cofactor essential in permitting continued glycolytic activity 1. The HIF-dependent up-regulation of PDK1 in *Phd2* deficient neurons had, however, only a very modest effect on the steady-state level of phosphorylated PDHA1 (Figure 2C). Interestingly, genetic inactivation of *Hif1a* and *Hif2a* significantly reduced the net phosphorylation of PDHA1 suggesting that residual HIF-1 activity under normoxic conditions may contribute to the regulation of basal PDH complex activity in neurons (Figure 2C). Elevated lactate and PDK1 protein levels were also confirmed in brains of *Phd2* deficient mice *in vivo* (Figure 2D-E). Moreover, in the presence of dichloroacetate (DCA), a non-selective PDK inhibitor, enhanced lactate production in *Phd2* deficient neurons was mostly prevented, whereas increased glucose consumption was only partially reversed (Figure 2F). Importantly, neuronal cell death in response to ischemic stress conditions *in vitro* was significantly alleviated in *Phd2* deficient neurons (Figure 2G). Exposure to DCA prior to OGD, however, fully reversed the increased ischemic tolerance in* Phd2* deficient neurons (Figure 2G). Overall, our results suggest that HIF-dependent metabolic reprogramming toward aerobic glycolysis, as the main source for ATP, due to genetic inactivation of PHD2 improves neuronal ischemic tolerance.

**Figure 3:**
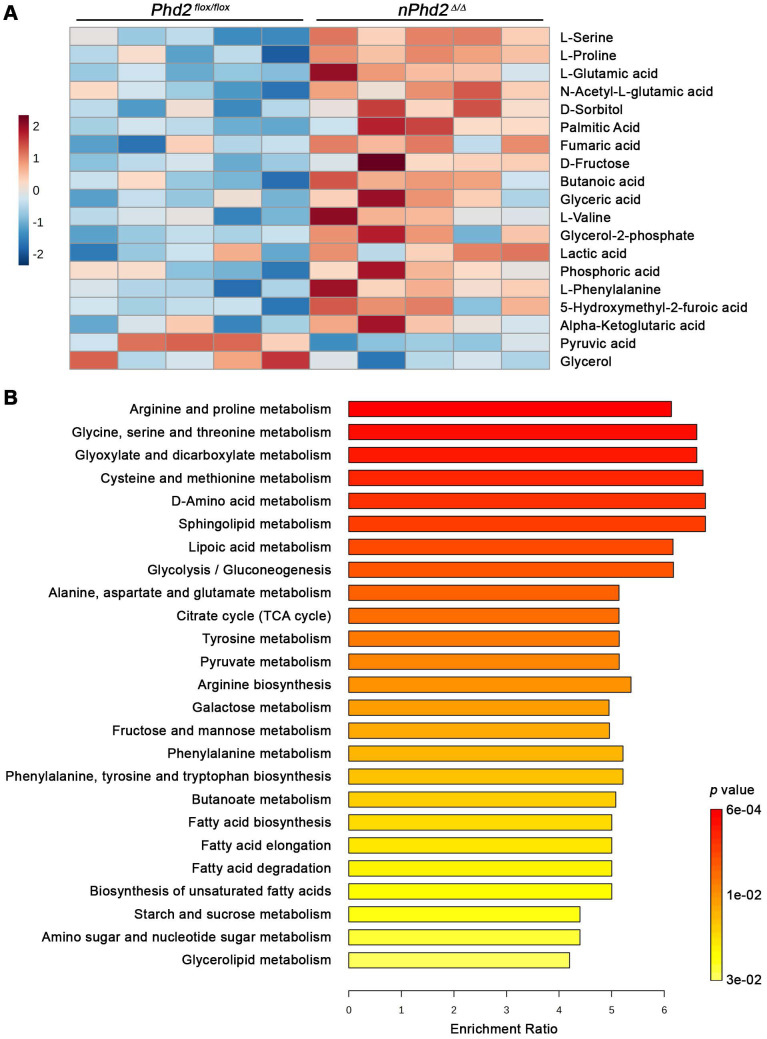
** Neuron-specific PHD2 inactivation leads to substantial changes in metabolite levels related to the brain central carbon metabolism.** GC/MS technique was used for metabolic profiling of brain tissue samples derived from adult *Phd2 ^f/f^* and *nPhd2 ^Δ/Δ^* mice. **(A)** Heat map showing 19 significantly differential metabolites in the brain of *nPhd2 ^Δ/Δ^* mice compared to *Phd2 ^f/f^* littermates (*n* = 5 per group; unpaired two-tailed Student's *t* test; * *p* < 0.05). **(B)** Quantitative pathway enrichment analysis of differential metabolites conducted using MetaboAnalyst 6.0 (*p*-values from pathway enrichment analysis are plotted on the y-axis, and pathway impact values from pathway topology analysis on the x-axis). The enrichment ratio plotted on the x-axis is calculated as the number of hits within a particular metabolic pathway divided by the expected number of hits. The top 25 enriched pathways are shown.

Moreover, we conducted semi-targeted GC/MS to estimate the impact of neuronal *Phd2* deficiency on the CNS metabolism *in toto*. Among the 73 detected metabolites, 17 metabolites (e.g. alpha-ketoglutaric acid, L-glutamic acid, lactic acid, fumaric acid) were significantly elevated and 2 metabolites (pyruvic acid, glycerol) were significantly decreased in the brain of *nPhd2 ^Δ/Δ^* mice as compared to *Phd2 ^f/f^* littermates (Figure 3A, Table S7). The differential metabolites were used for quantitative enrichment analysis. A total of 29 metabolic pathways were shown to be enriched, of which nine are related to carbohydrate metabolism (glycolysis/gluconeogenesis; pentose phosphate pathway; pyruvate metabolism; TCA cycle; amino sugar and nucleotide sugar metabolism; glyoxylate and dicarboxylate metabolism; fructose and mannose metabolism; galactose metabolism; starch and sucrose metabolism), eleven related to biosynthesis and degradation of various amino acids, and even nine pathways related to lipid metabolism (Figure 3B, Table S8). These findings not only confirm that HIF-dependent processes affect the neuronal carbohydrate metabolism at many levels, but also provide evidence that HIF signaling exert pleiotropic effects on the amino acid and lipid metabolism.

### Systemic treatment with roxadustat affects the central carbon and amino acid metabolism in murine brain

**Figure 4:**
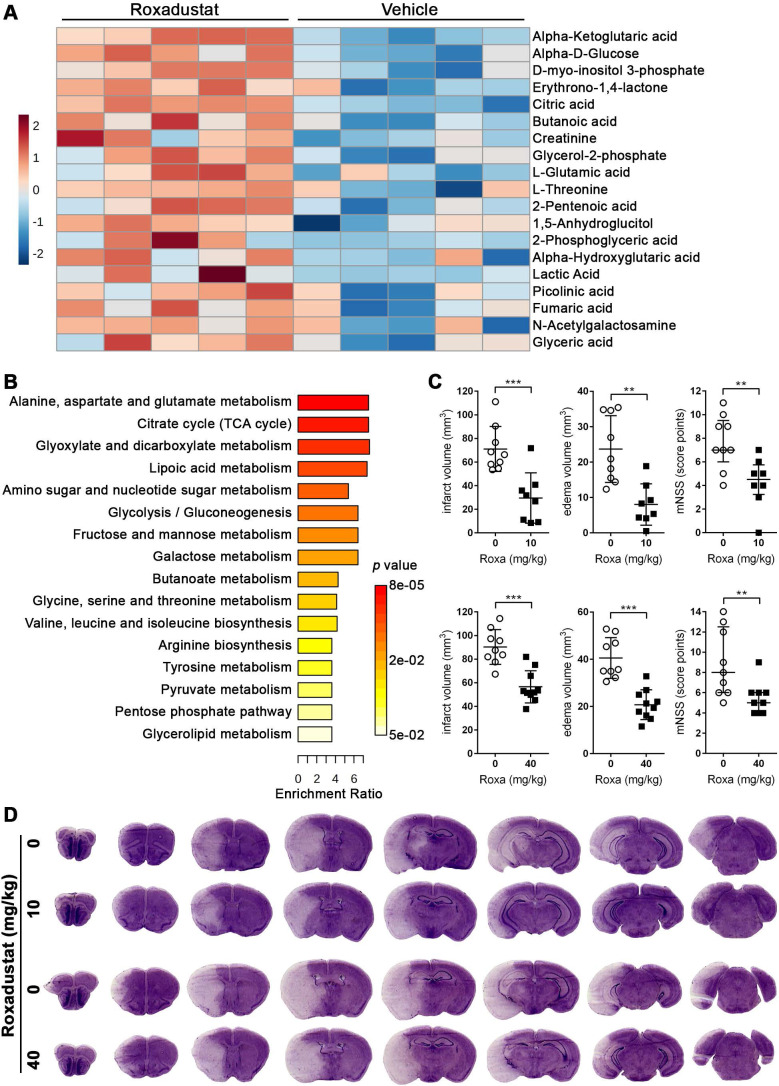
** Systemic treatment with the HIF-PHI roxadustat affects the brain metabolism and improves the outcome from acute ischemic stroke. (A, B)** Roxadustat was applied intraperitoneally to C57BL/6 mice at a dosage of 40 mg/kg body weight twice daily with 12 h between doses for 4 days. Control mice received equal volumes of vehicle solution. GC/MS technique was applied for metabolic profiling of brain tissue samples. **(A)** Heat map showing 19 significantly differential metabolites in the brain of roxadustat treated mice compared to vehicle controls (*n* = 5 per group; unpaired two-tailed Student's *t* test; * *p* < 0.05). **(B)** Quantitative pathway enrichment analysis of differential metabolites conducted using MetaboAnalyst 6.0 (*p*-values from pathway enrichment analysis are plotted on the y-axis, and pathway impact values from pathway topology analysis on the x-axis). The enrichment ratio plotted on the x-axis is calculated as the number of hits within a particular metabolic pathway divided by the expected number of hits. **(C)** Upon treatment with roxadustat (10 mg/kg or 40 mg/kg twice daily for 4 days) or vehicle solution, mice were subjected to 60 min of MCAO followed by 24 h reperfusion. Infarct and edema volume were determined by cresyl violet staining (*n* = 8-10 per group; unpaired two-tailed Student's *t* test; ** *p* < 0.01, *** *p* < 0.001). Neurological function was assessed using the modified neurological severity score (median with interquartile range; *n* = 8-10 per group; Mann-Whitney *U* rank-sum test; ** *p* < 0.01). **(D)** Representative images of cresyl violet-stained coronal brain sections from vehicle and roxadustat treated animals subjected to experimental ischemic stroke.

To evaluate the potential relevance for stroke therapy, mice were systemically treated with roxadustat, a low-molecular weight HIF-PHD inhibitor (HIF-PHI) approved in a number of countries for the treatment of symptomatic anemia associated with chronic kidney disease 38, and semi-targeted GC/MS was applied to determine potential changes in brain metabolism. Among the 79 detected metabolites, 18 metabolites were significantly increased upon systemic roxadustat treatment (Figure 4A, Table S9). A comparison of the significant differential metabolites revealed six metabolites (lactic acid, alpha-ketoglutaric acid, L-glutamic acid, fumaric acid, glycerol-2-phosphate and glyceric acid) overlapping between roxadustat treated mice and *Phd2* deficient animals. Further quantitative MSEA uncovered 16 significantly enriched metabolic pathways entirely overlapping with those identified in the CNS of *Phd2* deficient animals. (Figure 4B, Table S10). Among them are eight carbohydrate metabolism-related pathways (glycolysis/gluconeogenesis; pentose phosphate pathway; pyruvate metabolism; TCA cycle; amino sugar and nucleotide sugar metabolism; glyoxylate and dicarboxylate metabolism; fructose and mannose metabolism; galactose metabolism), five amino acid metabolism-related pathways (arginine biosynthesis; alanine, aspartate and glutamate metabolism; glycine, serine and threonine metabolism; valine, leucine and isoleucine biosynthesis; tyrosine metabolism), as well as three pathways related to lipid metabolism (lipoic acid metabolism; butanoate metabolism; glycerolipid metabolism) (Figure 4B, Table S10). In summary, either genetic or pharmacological activation of the HIF signaling pathway lead to considerable and highly comparable changes in the central carbon metabolism of the CNS.

### Preventive roxadustat treatment ameliorates cerebral tissue damage and sensorimotor dysfunction after acute ischemic stroke

Next, we investigated if a preventive treatment with roxadustat affects the histopathological and functional outcome from acute ischemic stroke. Mice that received either a low or high dose of roxadustat systemically prior to ischemic stroke evolved markedly reduced infarct lesions and vasogenic brain edema as compared to vehicle treated animals (Figure 4C-D), whereas post-stroke weight loss and mortality were comparable between all groups (Figure S2). Moreover, improved cerebral tissue damage was accompanied by a significantly decreased sensorimotor impairment of roxadustat treated mice (Figure 4C). As compared to sham-operated animals cerebral I/R injury caused substantial up-regulation of numerous pro-inflammatory factors including tumor necrosis factor alpha (*Tnf*), interleukin-1 beta (*Il1b*), interleukin-6 (*Il6*), monocyte chemoattractant protein-1 (*Mcp1*) and cyclooxygenase-2 (*Cox2*) (Figure S4A). However, transcript levels of pro-inflammatory cytokines were not significantly different between vehicle and roxadustat treated mice in both healthy and stroke-affected brain (Figure S4A) suggesting that systemic roxadustat treatment neither promotes inflammation in the intact CNS nor affects the local pro-inflammatory response during acute ischemic stroke. Given that vascular endothelial growth factor A (VEGF-A), a key factor for the vascular response to cerebral ischemia, is a direct, bona fide HIF-1 target gene, we monitored cerebral cortical blood flow (CBF) during and after transient cerebral ischemia *in vivo*. High-resolution laser speckle contrast imaging (LSCI) confirmed a substantial reduction of CBF across the peri-infarct area (-40%), and even stronger within the infarct core (-60%) upon MCAO (Figure S4B). CBF in the ipsilateral brain hemisphere during MCAO was, however, quite similar in vehicle and roxadustat treated mice (Figure S4B). Interestingly, cortical perfusion of the non-affected brain hemisphere was significantly lower in roxadustat treated mice as compared to animals receiving vehicle (Figure S4B). MCA re-opening provoked a gradual reperfusion of infarct core and peri-infarct area, albeit not completely when compared to the CBF in the contralateral hemisphere 3 h after onset of reperfusion (Figure S4B). The reperfusion of either entire ipsilateral hemisphere, peri-infarct area or infarct core region was not significantly affected by roxadustat pre-treatment (Figure S4B). Accordingly, systemic roxadustat treatment did not enhance the expression of *Vegfa* in both intact CNS and stroke-affected brain (Figure S4A). Overall, our results suggest that the improved outcome from cerebral ischemia in mice receiving the HIF-PHI roxadustat before stroke does not involve changes in the inflammatory response, cerebral blood flow or brain vasculature remodeling, but is the result of reprogramming of brain metabolism.

### Roxadustat increases the expression of HIF-targeted glucose transporter and glycolytic enzymes in murine brain cells

**Figure 5:**
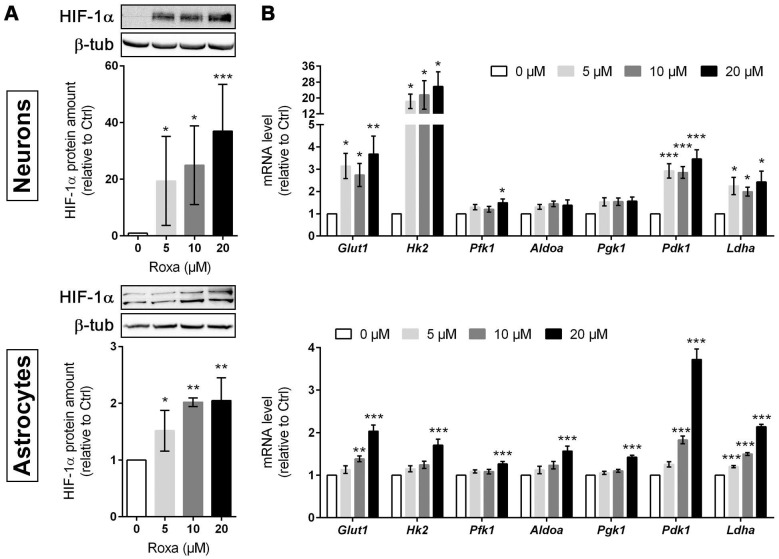
** Roxadustat increases the expression of HIF-targeted glucose transporter and glycolytic enzymes.** Primary neurons and astrocytes were treated with roxadustat for 6 h or 24 h. DMSO treated cells were used as control (Ctrl).** (A)** HIF-1α protein levels were quantified by Western blotting. Values are normalized to β-tubulin and expressed as fold change of Ctrl (*n* = 3-6 per group; One-way ANOVA with Holm-Sidak's multiple comparisons test; * *p* < 0.05, ** *p* < 0.01, *** *p* < 0.001).** (B)** Real-time RT-PCR was used to determine transcript levels of HIF target genes involved in glucose metabolism. Values are normalized to *Rps12* and expressed as fold change of Ctrl (*n* = 6-9 per group; One-way ANOVA with Holm-Sidak's multiple comparisons test; * *p* < 0.05, ** *p* < 0.01, *** *p* < 0.001).

To decipher potential HIF-dependent metabolic changes at a cellular level, we studied the glucose metabolism in primary murine neurons and astrocytes, which together account for the majority of brain-resident cells, exposed to roxadustat *in vitro*. Pre-experiments revealed cytotoxic effects of roxadustat on neurons at concentrations of ≥50 µM (Figure S5A), whereas the viability of astrocytes remained unaffected by roxadustat applied at a dose of 5 to 100 µM (Figure S5B). In both neurons and astrocytes, roxadustat significantly increased the amount of HIF-1α protein in a dose-dependent manner (Figure 5A). Accordingly, the expression of certain HIF target genes, whose function is related to glucose metabolism, was substantially increased upon roxadustat treatment. In neurons, mRNA levels of *Glut1* and glycolytic enzymes including *Hk2*, *Pfk1*, and *Ldha* were significantly enhanced (Figure 5B). Similarly, roxadustat significantly up-regulated *Glut1*, *Hk2*, *Pfk1*, *Aldoa*, *Pgk1*, and *Ldha* transcript levels in astrocytes (Figure 5B). These results suggest that pharmacological HIF activation through the PHI roxadustat increases glucose uptake and glycolytic flux in murine brain cells.

### Roxadustat enhances glycogen synthesis and promotes a metabolic shift to increased glycolytic energy production in neurons and astrocytes

**Figure 6:**
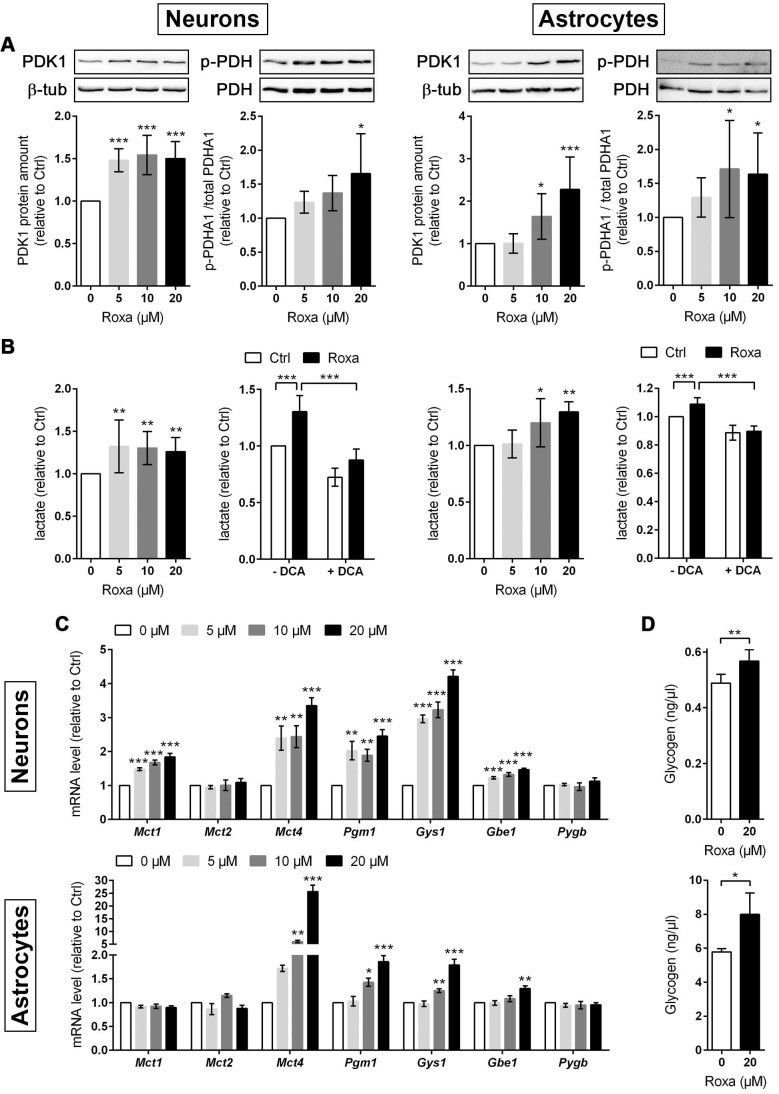
** Roxadustat enhances pyruvate-to-lactate flux through PDK1-mediated inhibition of the pyruvate dehydrogenase, increases lactate transport capacity as well as promotes glycogen synthesis.** Neurons and astrocytes were treated with roxadustat in the absence or presence of 5 mM DCA for 6 h or 24 h. DMSO treated cells were used as control (Ctrl). **(A)** PDK1, p-PDHA1 and total PDHA1 protein levels were quantified by Western blotting. Values are normalized to β-tubulin and expressed as fold change of Ctrl (*n* = 6-7 per group; One-way ANOVA with Holm-Sidak's multiple comparisons test; * *p* < 0.05, *** *p* < 0.001).** (B)** Lactate production was determined in cell culture supernatants using an enzymatic assay. Values are expressed as fold change of Ctrl (*n* = 5-10 per group; One- or Two-way ANOVA with Holm-Sidak's multiple comparisons test; * *p* < 0.05, ** *p* < 0.01, *** *p* < 0.001).** (C)** Real-time RT-PCR was used to determine transcript levels of monocarboxylate transporter (MCT) isoforms. Values are normalized to *Rps12* and expressed as fold change of Ctrl (*n* = 5 per group; One-way ANOVA with Holm-Sidak's multiple comparisons test; ** *p* < 0.01, *** *p* < 0.001).

Furthermore, mRNA and protein levels of PDK1 were significantly increased in neurons and astrocytes upon roxadustat treatment (Figure 5B and 6A). Along with the up-regulation of PDK1, neurons and astrocytes responded to roxadustat with significantly enhanced PDHA1 phosphorylation and extracellular lactate levels (Figure 6A-B). Accordingly, DCA prevented the increased lactate production in roxadustat treated neurons and astrocytes (Figure 6B).

A high glycolytic activity cannot be sustained without a matching and increased lactate excretion into the extracellular space. In brain-resident cells lactate fluxes are predominantly mediated by the proton-linked monocarboxylate transporter (MCT) isoforms MCT1, 2 and 4 39. In accordance to the increased extracellular lactate levels (Figure 6B), roxadustat significantly increased the expression of *Mct1* and *Mct4* in neurons, while *Mct2* mRNA levels were not altered (Figure 6C). Similarly, astrocytes exhibited a substantial upregulation of *Mct4*, whereas *Mct1* and *Mct2* transcript levels remained constant upon roxadustat treatment (Figure 6C). As *Phd2* depleted neurons also showed significantly elevated expression of *Mct1* and *Mct4* (Figure S6A), our results suggest that activation of HIF-1 in neurons and glial cells not only upregulates glycolytic lactate production, but also facilitates its efficient excretion. Despite having different affinities for lactate, MCTs are equilibrative lactate/H^+^ transporters, whereas the rate of transport in either direction depends on the prevailing gradients of substrate and pH 39. Thus, HIF-1-dependent upregulation of MCT1 and MCT4 may also promote bidirectional lactate exchange between glial cells and neurons under certain circumstances, as proposed by the so-called astrocyte-to-neuron lactate shuttle (ANLS) hypothesis 40. Moreover, neurons and astrocytes exposed to roxadustat elicited significantly increased expression of central enzymes in glycogen biosynthesis including phosphoglucomutase 1 (*Pgm1*), glycogen synthase (*Gys1*) and 1,4-alpha-glucan branching enzyme 1 (*Gbe1*), whereas transcript levels of the enzyme glycogen phosphorylase B (*Pygb*), which catalyzes the first step of glycogenolysis, remained stable during treatment (Figure 6C). Similar to the pharmacological approach, genetic activation of HIF-1 in neurons caused robust up-regulation of glycogen-biosynthesis enzymes (Figure S6A). Accordingly, the cellular glycogen content of roxadustat treated neurons and astrocytes was elevated by 16% and 38%, respectively (Figure 6D).

**Figure 7:**
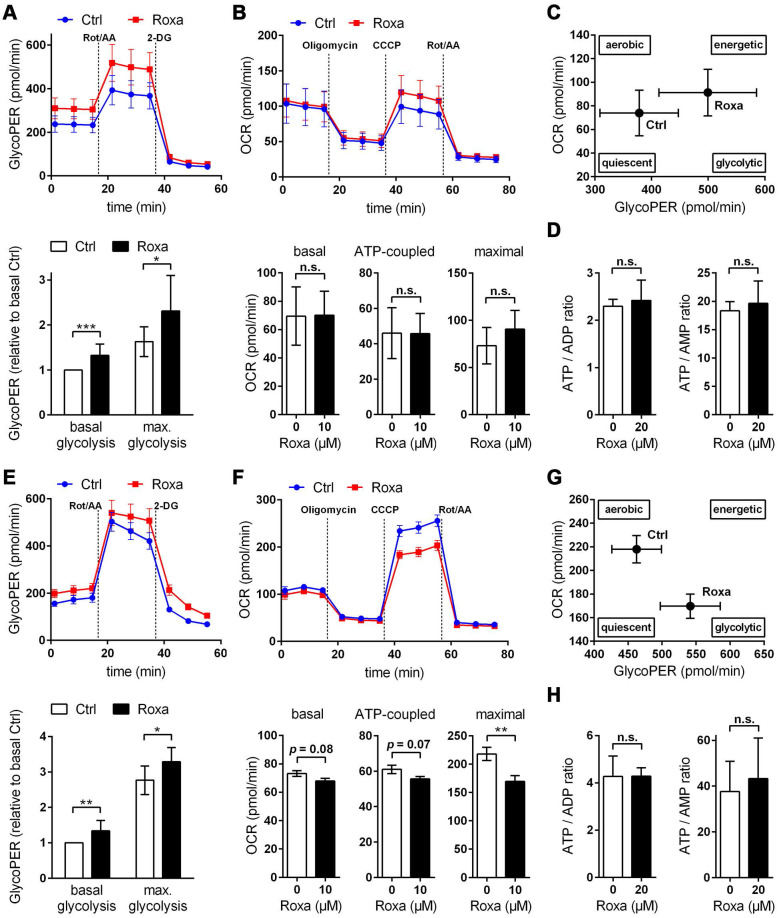
** Roxadustat promotes a metabolic shift to increased glycolytic energy production.** The bioenergetic state of **(A-C)** neurons and **(E-G)** astrocytes treated with 10 µM roxadustat for 24 h were determined using a Seahorse XF96e Analyzer. DMSO treated cells were used as control (Ctrl). **(A, E)** Cell glycolytic profile was evaluated by using the Glycolytic Rate Assay. Proton efflux rate (PER) and oxygen consumption rate (OCR) were measured under basal conditions and in response to rotenone/antimycin A and 2-deoxy-D-glucose (2-DG). Glycolytic PER (GlycoPER) was calculated to determine basal glycolysis and compensatory glycolysis (in response to Rot/AA) (*n* = 6-12 per group; unpaired two-tailed Student's *t* test; * *p* < 0.05, ** *p* < 0.01, *** *p* < 0.001).** (B, F)** Cell Mito Stress Test was applied to estimate key parameters of mitochondrial respiration. OCR was measured after sequential addition of oligomycin, CCCP, and Rot/AA to calculate basal respiration (= OCR_basal_ - OCR_Rot/AA_), ATP production-linked respiration (= (OCR_basal_ - OCR_Oligo_) - OCR_Rot/AA_) and maximal respiration (= OCR_CCCP_ - OCR_Rot/AA_) (*n* = 8-11 per group; unpaired two-tailed Student's *t* test; ** *p* < 0.01). **(C, G)** Energy Map depicting the relative bioenergetic state of Ctrl and roxadustat treated cells.** (D, H)** Cellular AMP, ADP and ATP levels were measured by LC-MS/MS technique and the ATP/ADP and ATP/AMP ratios were calculated (*n* = 4-7 per group; unpaired two-tailed Student's *t* test).

For a detailed overview of the brain cell energy metabolism upon pharmacological activation of the HIF signaling pathway, we next conducted Seahorse extracellular flux analysis allowing continuous direct quantification of mitochondrial respiration and glycolysis. Firstly, we used the glycolysis rate assay to determine the rate of basal glycolysis and of compensatory glycolysis following inhibition of the complex I and III of the mitochondrial ETC by rotenone (Rot) and antimycin A (AA), respectively. This assay utilizes both proton efflux rate (PER) and oxygen consumption rate (OCR) measurements to determine the glycolytic proton efflux rate (glycoPER), discounting the effect of mitochondrial-derived CO_2_ on extracellular acidification. In accordance to the increased lactate production shown in Figure 6B, roxadustat significantly elevated the basal glycolysis in neurons and astrocytes (Figure 7A and 7E). In both neurons and astrocytes roxadustat also significantly increased the compensatory glycolysis determined upon administration of Rot/AA shifting the burden of ATP supply to meet the cells' energy demands entirely from OXPHOS to glycolysis (Figure 7A and 7E). Secondly, we used the mitochondrial stress test to assess basal respiration, ATP production-linked respiration and maximal mitochondrial respiration upon sequential application of oligomycin (inhibits complex V (ATP-synthase) of the ETC) and CCCP (uncoupling agent that collapses the proton gradient resulting in maximum oxygen consumption by complex IV). In neurons basal, ATP production-coupled and maximum respiration were not affected by roxadustat (Figure 7B). In roxadustat treated astrocytes basal and ATP production-linked respiration were non-significantly reduced, whereas the maximal respiratory capacity was significantly decreased (Figure 7F). Further, maximal rates for both OCR and GlycoPER were plotted against one another to create a cell's ''energy map'' that uncovers potential shifts in metabolic programs with increased aerobic energy production appearing as a positive shift along the y-axis and increased glycolytic energy production appearing as a positive shift along the x-axis. Positive shifts along both axes represent a heightened energetic state, whereas negative shift along either axis indicates a quiescent state. In neurons roxadustat activated an energetic phenotype with an increased glycolytic energy production as the main driver of the heightened energetic state (Figure 7C). In astrocytes roxadustat shifted the energy production from mitochondrial OXPHOS to glycolysis (Figure 7G). In order to further characterize the impact of roxadustat on the baseline energy status of neurons and glial cells, intracellular levels of ATP, ADP and AMP were quantified. Total ATP, ADP and AMP levels in neurons were increased by roxadustat treatment, whereas ATP/ADP and ATP/AMP ratio as well as the adenylate energy charge were comparable between roxadustat treated and control cells (Table S11, Figure 7D, Figure S5C). In astrocytes roxadustat did not affect ATP, ADP and AMP levels, ATP/ADP and ATP/AMP ratio and the adenylate energy charge (Table S11, Figure 7H, Figure S5D). Overall, these results imply that pharmacological activation of the HIF-1 signaling pathway in brain-resident cells alleviates the capacity for oxygen-independent glycolytic energy production through increasing glucose uptake, glycolytic flux, redirection of glucose-derived pyruvate flux into lactate, and lactate secretion. Beyond, activation of HIF signaling promoted glycogen biosynthesis suggesting a prolonged maintenance of energy generation in brain-resident cells when blood glucose supply is restricted.

### Pharmacological HIF activation enhances the ischemic tolerance of astrocytes, but not neurons, through glucose metabolism reprogramming

**Figure 8:**
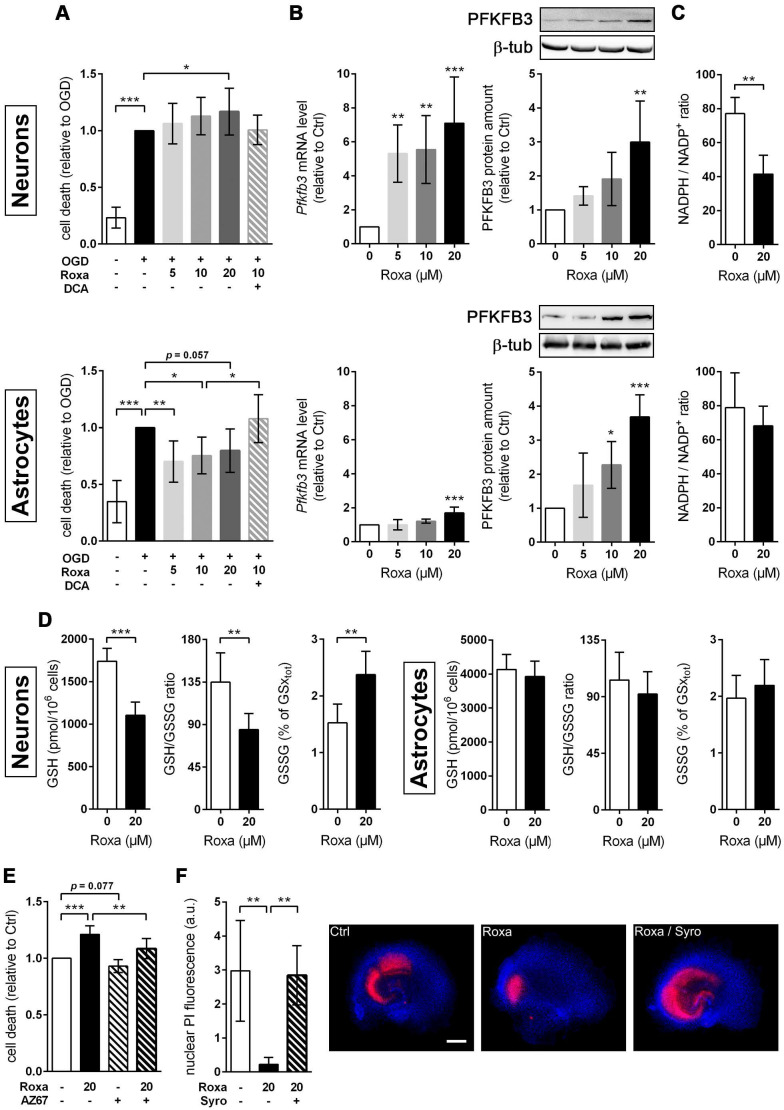
** Roxadustat lowers the vulnerability of brain-resident cells towards ischemic stress through glucose metabolism reprogramming. (A)** Neurons and astrocytes were treated with roxadustat in the absence or presence of 5 mM DCA for 24 h prior to exposure to OGD conditions (glucose-free aCSF; 0.2% O_2_) for 4 h. DMSO pre-treated cells exposed to aCSF (+Glc) under normoxic conditions for 4 h are used as control (Ctrl). Cell viability was determined using a dead-cell protease activity assay. Values are expressed as fold change of OGD-subjected DMSO pre-treated cells (*n* = 7-12 per group; One-way ANOVA with Holm-Sidak's multiple comparisons test; * *p* < 0.05, ** *p* < 0.01, *** *p* < 0.001). **(B-D)** Neurons and astrocytes were treated with roxadustat for 6 h or 24 h. DMSO treated cells were used as Ctrl. **(B)** Real-time RT-PCR and Western blotting were applied to determine transcript and protein levels of PFKFB3. Values are normalized to *Rps12* (mRNA) or β-tubulin (protein) and expressed as fold change of Ctrl (*n* = 4-9 per group; One-way ANOVA with Holm-Sidak's multiple comparisons test; ** *p* < 0.01, *** *p* < 0.001). **(C)** Cellular NADP^+^ and NADPH levels were measured by LC-MS/MS technique and the NADPH/NADP^+^ ratio was calculated (*n* = 4-7 per group; unpaired two-tailed Student's *t* test; ** *p* < 0.01). **(D)** Levels of total (GSx_tot_), reduced (GSH), and oxidized (GSSG) glutathione were quantified by UPLC and the ratios of GSH to GSSG and GSSG to GSx_tot_, respectively, were calculated (*n* = 6 per group; unpaired two-tailed Student's *t* test; ** *p* < 0.01, *** *p* < 0.001). **(E)** Neurons were treated with roxadustat and/or 10 nM AZ-PFKFB3-67 (AZ67) for 24 h and 4 h, respectively, prior to exposure to OGD conditions (glucose-free aCSF; 0.2% O_2_) for 4 h. DMSO pre-treated cells exposed to OGD conditions for 4 h are used as Ctrl. Cell viability was determined using a dead-cell protease activity assay. Values are expressed as fold change of Ctrl (*n* = 6 per group; One-way ANOVA with Holm-Sidak's multiple comparisons test; ** *p* < 0.01, *** *p* < 0.001). **(F)** OHSCs were treated with 10 µM syrosingopine (Syro) and/or roxadustat for 24 h before exposure to OGD (glucose-free aCSF; 0.2% O_2_) for 30 min followed by 24 h of reoxygenation. DMSO pre-treated OHSCs exposed to OGD/R are used as Ctrl. Cellular uptake of propidium iodide (PI) was used to estimate neuronal cell death within the hippocampal CA sub region. (*n* = 5-6 per group; One-way ANOVA with Holm-Sidak's multiple comparisons test; ** *p* < 0.01). Representative microphotographs: PI (red), DAPI (blue). Scale bar, 500 µm.

Further, we investigated whether the roxadustat-induced metabolic reprogramming influences the vulnerability of brain cells towards ischemic stress. Exposure to OGD strongly diminished the viability of both neurons and astrocytes (Figure 8A-B). Contrary to the PHD2-specific genetic approach (Figure 2G), non-selective pharmacological inhibition of all three PHD isoenzymes with roxadustat increased neuronal cell death upon OGD in a dose-dependent manner (Figure 8A). By contrast, the cell death in OGD-exposed astrocytes, pre-treated with roxadustat, was significantly reduced (Figure 8B). Moreover, the cytoprotective effect of roxadustat on astrocytes was fully abrogated by the addition of DCA (Figure 8B). Collectively, our data indicate that pharmacological activation of the HIF signaling pathway selectively increases the ischemic tolerance of astrocytes at least in part by a PDK-dependent reprogramming of the glucose metabolism.

### Roxadustat impairs the neuronal redox balance through a PFKFB3-dependent reduction of NADPH production in the pentose phosphate pathway

Cumulative evidence indicates that neurons have a lower glycolytic rate than astrocytes, and a significant proportion of glucose-6-phosphate (G6P) is directed towards the pentose-phosphate pathway (PPP) 41, 42. A major function of the PPP is the formation of NADPH, a necessary cofactor for the reduction of glutathione disulfide, to provide neuroprotection from oxidative stress. Preferential glucose consumption through the PPP is achieved by inhibiting glycolysis via continuous degradation of the glycolytic positive-effector protein, 6-phosphofructo-2-kinase/fructose-2,6-bisphosphatase-3 (PFKFB3) 41, 42.

In astrocytes, however, despite considerable PPP activity, there is substantial metabolism of glucose by glycolysis due in part to higher basal expression of both glucose-6-phosphate dehydrogenase (G6PD), catalyzing the rate-limiting step of the PPP, and PFKFB3 when compared with neurons 41, 42. Interestingly, it has been shown in various cell types and organs that PFKFB3 is upregulated by hypoxia or hypoxia mimics in a HIF-1-dependent manner 43-45. Considering these facts, we hypothesized that roxadustat causes redirection of G6P from PPP to the glycolysis pathway by HIF-1-dependent upregulation of PFKFB3, which may further decrease the low intrinsic antioxidant capacity of neurons, making them more susceptible to ischemic stress. In accordance to the activation of HIF-1 (Figure 5A), roxadustat significantly increased the PFKFB3 expression on mRNA and protein level in a dose-dependent manner in both neurons and astrocytes (Figure 8B). Interestingly, in *Phd2* deficient neurons showing a comparatively less pronounced increase of HIF-1α protein abundance (data not shown), PFKFB3 protein levels remained stable, while transcript levels were slightly but significantly increased (Figure S6B) suggesting that up-regulation of PFKFB3 expression, which exceeds the constant proteasomal degradation of PFKFB3 in neurons, may require strong activation of HIF-1. Despite comparable upregulation of PFKFB3 in roxadustat treated neurons and astrocytes, a significantly increased NADP^+^ level and a substantially lowered NADPH/NADP^+^ ratio was only found in neurons, but not in astrocytes (Table S11, Figure 8C). Accordingly, roxadustat treatment significantly increased the portion of oxidized glutathione (GSSG) as well as decreased the amount of reduced glutathione (GSH) and the GSH/GSSG ratio in neurons, while it did not affect the glutathione redox system in astrocytes (Figure 8D). It suggests that roxadustat-induced HIF-dependent metabolic reprogramming *in toto* have both beneficial and detrimental effects on the ischemic tolerance of neurons. While HIF-dependent elevation of glucose uptake, glycogen stores and glycolytic flux might be useful to sustain neuronal ATP production under hypoxic/ischemic conditions, compromising the cytoplasmic production of NADPH via hijacking glucose flux from PPP through PFKFB3 further lowers the weak intrinsic antioxidant reserve of neurons, making them more prone to ischemic stress. Indeed, co-treatment with AZ67, a selective PFKFB3 inhibitor, significantly reversed the enhanced cell death of roxadustat treated neurons exposed to OGD (Figure 8E).

### Roxadustat-mediated neuroprotection in organotypic hippocampal cultures exposed to ischemic stress is prevented by restricting glycolysis through lactate transport blockade

Considering the improved histopathological and functional outcome of mice treated with roxadustat prior to ischemic stroke (Figure 4C), we analyzed whether roxadustat protects neurons in organotypic slice cultures of the murine hippocampus against ischemia-induced cell death. In contrast to monolayer cultures of dissociated neurons, organotypic slice cultures maintain the cytoarchitecture of a specific brain region with appropriate neuronal glial relationship, and, thus, provide an excellent *ex vivo* model system for investigating glia-neuron interplay under physiological and pathophysiological conditions. In OHSCs, treatment with 20 µM roxadustat prior to an OGD/reoxygenation insult significantly decreased the cell death of CA pyramidal neurons, known to be highly vulnerable to ischemia (Figure 8F). In order to analyze if HIF-dependent metabolic shift toward glycolytic energy production contributes to the neuroprotective effects elicited by preventive roxadustat treatment, OHSCs were co-treated with roxadustat and 10 µM syrosingopine (Su-3118), a dual MCT1/MCT4 inhibitor that hampers glycolysis by preventing lactate efflux. Indeed, MCT1/MCT4 inhibition largely prevented the roxadustat-mediated protection of CA neurons from ischemic cell death (Figure 8F). Together, our findings suggest that a well-balanced HIF-dependent elevation of glucose uptake, glycogen synthesis, glycolytic capacity, and lactate release prior to cerebral ischemia improves the structural and functional integrity of brain-resident cells.

## Discussion

In order to maintain energy and redox homeostasis under ischemia, the brain initiates intrinsic compensatory pathways, which among others comprise metabolic reprogramming strategies. We here demonstrate that sustained activation of the HIF pathway in the murine brain by administration of the pan-HIF-PHI roxadustat causes extensive reprogramming of carbohydrate, amino acid and lipid metabolism-related pathways. Given the substantially reduced tissue injury and functional impairments determined in mice systemically treated with roxadustat prior to ischemic stroke, we suppose that HIF-mediated metabolic reprogramming in brain-resident cells confers resistance to subsequent cerebral ischemia.

To sustain fuel oxidation by the TCA cycle, nerve cells use alternative energy substrates, which partly reduce the dependence of the brain on a continuous supply of blood glucose, therefore improving the brain's resistance to ischemia 46. Cells upregulate glutaminolysis, which produces α-ketoglutarate from glutamine that undergoes further oxidation in the TCA cycle for the purpose of energy generation 46. Moreover, a previous study has demonstrated that cerebral ischemia caused a ketogenic response as shown by enhanced hepatic free fatty acid β-oxidation and increased serum and brain β-hydroxybutyrate levels 47. Although most ketones are synthesized in the liver, astrocytes can also generate ketone bodies from fatty acid β-oxidation. All brain cell types including neurons are able to uptake and metabolize ketones to acetyl-CoA to support the cell energy 46. Accordingly, in rodents both L-glutamine administration and ketogenic diet provided protection against brain ischemic injury 48, 49.

In particular, astrocytes sense excitatory neuronal activity by taking up excess glutamate from the synaptic cleft. As glutamate uptake is an energy-consuming process, neurotransmission progresses with increased energy demands in astrocytes, which stimulate astrocytic glucose uptake from peripheral blood, aerobic glycolysis and lactate release mainly via MCT1, 2 and 4 50. Astrocyte-derived lactate is proposed to be transferred to neurons through MCTs, where it is primarily used, after conversion to pyruvate, as a rapid energy substrate, known as the ANLS hypothesis 50. Considering the rapid and massive release of glutamate from neurons as a result of energy failure in cerebral ischemia, ANLS may provide an intrinsic compensatory mechanism to ameliorate bioenergetic crisis in neurons during ischemic stroke. However, the ANLS hypothesis is still under debate as convincing experimental evidences for a directed transfer of lactate from astrocytes to neighboring neurons *in vivo* are still missing. Moreover, given that mitochondrial OXPHOS is mostly impaired during ischemia, it remains questionable whether astrocyte-derived lactate can be used by neurons to maintain their energy homeostasis before disinhibition of OXPHOS upon reoxygenation. Thus, it remains open whether HIF-dependent upregulation of glucose uptake, glycolysis and lactate excretion in astrocytes as demonstrated in the present study fuels ANLS potentially protecting neurons from ischemic insult. Nevertheless, a previous study demonstrated that intracerebroventricular or intravenous administration of lactate at the onset of reperfusion reduces brain damage and neurobehavioral deficits in a mouse model of ischemic stroke 51. Moreover, addition of exogenous lactate improved the cell survival in neuronal cultures exposed to OGD stress conditions 52. Interestingly, the use of other metabolic substrates, such as pyruvate or acetate, did not protect OGD-exposed neurons 53 suggesting that lactate-mediated post-ischemic neuroprotection cannot be solely attributed to its proposed role as a metabolic fuel for neurons, but may also involves other unknown complementary mechanisms.

Glycogen, predominantly located within astrocytes but also present in small amounts in neurons, is an important energy reserve in the adult brain and is believed to supply fuel during energy crisis 54-56. Accordingly, enhancement of astrocytic glycogenolysis improved the outcome from ischemic stroke *in vivo* as well as increased the survival of neurons in astrocyte-neuron co-cultures exposed to OGD *in vitro* 57. Neurons isolated from brain-specific muscle glycogen synthase deficient mice showed an increased susceptibility to hypoxia-induced cell death *in vitro* 56. Furthermore, pretreatment of rats with the HIF-PHI enarodustat protected the kidneys from ischemia by increasing glycogen storage in the kidneys via HIF-1-dependent upregulation of genes involved in glycogen synthesis 58. Accordingly, we here demonstrated that activation of HIF-1 resulted in increased glycogen storage through upregulation of key enzymes of glycogen synthesis in both astrocytes and neurons. In times of glucose availability, increasing glucose uptake to fuel glycogen stores together with enhancing the capacity for oxygen-independent energy production via glycolysis appears to be a powerful mechanism to maintain energy homeostasis in brain-resident cells when oxygen and glucose availability is limited as it occurs during ischemic stroke.

Compared with astrocytes, forebrain neurons have a weak intrinsic antioxidant reserve making them more vulnerable to oxidative stress. Bell et al. demonstrated that both basal and induced activity of transcription factor NF-E2-related factor 2 (Nrf2), a master regulator of antioxidant defense controlling a battery of antioxidant and detoxification genes, in maturing cortical neurons is extremely low due to epigenetic repression of the Nrf2 gene promoter 59. This seems paradoxical, since neurons produce more ROS than most cell types because of high metabolic activity, and post-mitotic central neurons apparently survive for many decades without being rendered dysfunctional by accumulating oxidative damage 59, 60. However, developmental inactivation of the Nrf2 pathway provides a more flexible redox environment, which is crucial for key redox-sensitive signaling pathways that mediate early neuronal development 59.

Nevertheless, neurons receive strong antioxidant support from surrounding glial cells, particularly astrocytes. Astrocytes have a high capacity for the Nrf2-driven production and storage of GSH, and release it into the extracellular space via the transporter multidrug resistance protein 1 in a manner that is increased in response to oxidative stress and chronically elevated levels of ambient glutamate, both hallmarks of excitotoxic disorders such as cerebral ischemia 60, 61. Astrocyte-derived GSH is broken down extracellularly and cysteine-containing products are taken up by neurons and used to synthesize their own GSH 60, 61. Accordingly, increasing GSH synthesis and release in astrocytes by overexpression or pharmacological activation of Nrf2 protected cocultured naive neurons from oxidative glutamate toxicity, whereas inhibition of glial GSH synthesis/release abolished glial-mediated neuronal protection 62. Moreover, neurons are also intrinsically equipped with a biochemical mechanism that links glucose metabolism to antioxidant defense. Neurons are programmed to metabolize a considerable proportion of glucose through the PPP generating NADPH, which maintains the antioxidant glutathione in its reduced state 50. This process is tightly controlled by the key glycolysis-promoting enzyme PFKFB3, which stimulates PFK1 activity by producing F2,6P2 (fructose 2,6-bisphosphate), the most potent allosteric activator of PFK1 50. In cortical neurons, the protein abundance of PFKFB3 is very low as it is constantly subjected to proteasome degradation after ubiquitylation by the E3 ubiquitin ligase APC/C (anaphase-promoting complex/cyclosome) and its adaptor Cdh1 50. In contrast, astrocytes express very low Cdh1 levels and therefore APC/C activity is neglectable in these cells leading to high PFKFB3 protein levels and a higher basal glycolytic rate 50. However, the preferential glucose utilization through PPP in neurons takes place at the expense of lowering the amount of glucose entering glycolysis for energy generation, which seems contradictory, in particular at the light that neurons constantly require energy to sustain glutamatergic neurotransmission 50. We here show that increased glycolytic flux in both roxadustat treated astrocytes and neurons was accompanied by enhanced PFKFB3 protein levels suggesting a redirection of the glucose flux away from PPP towards glycolysis. While HIF-dependent metabolic reprogramming in astrocytes decreased the susceptibility to ischemic stress, it, however, increased the vulnerability of neurons. In accordance to a reduced PPP activity, NADPH/NADP and GSH/GSSG ratios were substantially decreased in roxadustat treated neurons. Interestingly, despite comparable upregulation of PFKFB3 and glycolytic rate, NADPH/NADP and GSH/GSSG ratios in astrocytes were not affected by roxadustat treatment. This contradictory finding might be explained by the fact that astrocytes express about 10-fold more activity of the glutamate-cysteine ligase, rate-limiting enzyme for the GSH biosynthesis, and, thus, have a higher GSH reserve as mentioned earlier 41. Furthermore, the intrinsic PPP activity in astrocytes is five to seven times higher than that in neurons 63 suggesting that a subtle shunting of glucose away from the PPP toward glycolysis triggered by PFKFB3 severely impairs the intrinsic antioxidant capacity of neurons, but not that of astrocytes. Accordingly, we demonstrated that selective inhibition of PFKFB3 hampered the additional cytotoxic effect evoked by roxadustat in neuronal monocultures subjected to OGD. By contrast, moderate HIF activation by cell-specific genetic ablation of PHD2 in neurons triggered metabolic shift from OXPHOS towards aerobic glycolysis, but did not disturb the glucose flux to PPP by upregulation of PFKFB3, which ameliorated neuronal survival in *in vitro* and *in vivo* models of ischemic stroke. It suggests that augmented glucose import, glycogen storage and glycolytic capacity by activation of the HIF-1 pathway strengthen the intrinsic resistance of neurons to ischemic stress, whenever production of antioxidant NADPH through the PPP is maintained. Nevertheless, in our *ex vivo* and *in vivo* models, where neurons are able to interact bidirectionally with neighboring astrocytes, roxadustat treatment was sufficient to protect neurons from ischemic cell death. In the light of these findings, we suppose that HIF-1-induced reprogramming of the glucose metabolism (increasing glucose import, glycogen storage and glycolytic capacity) along with a sustained antioxidant support from surrounding astrocytes efficiently protects neurons from ischemic cell death.

**Figure 9:**
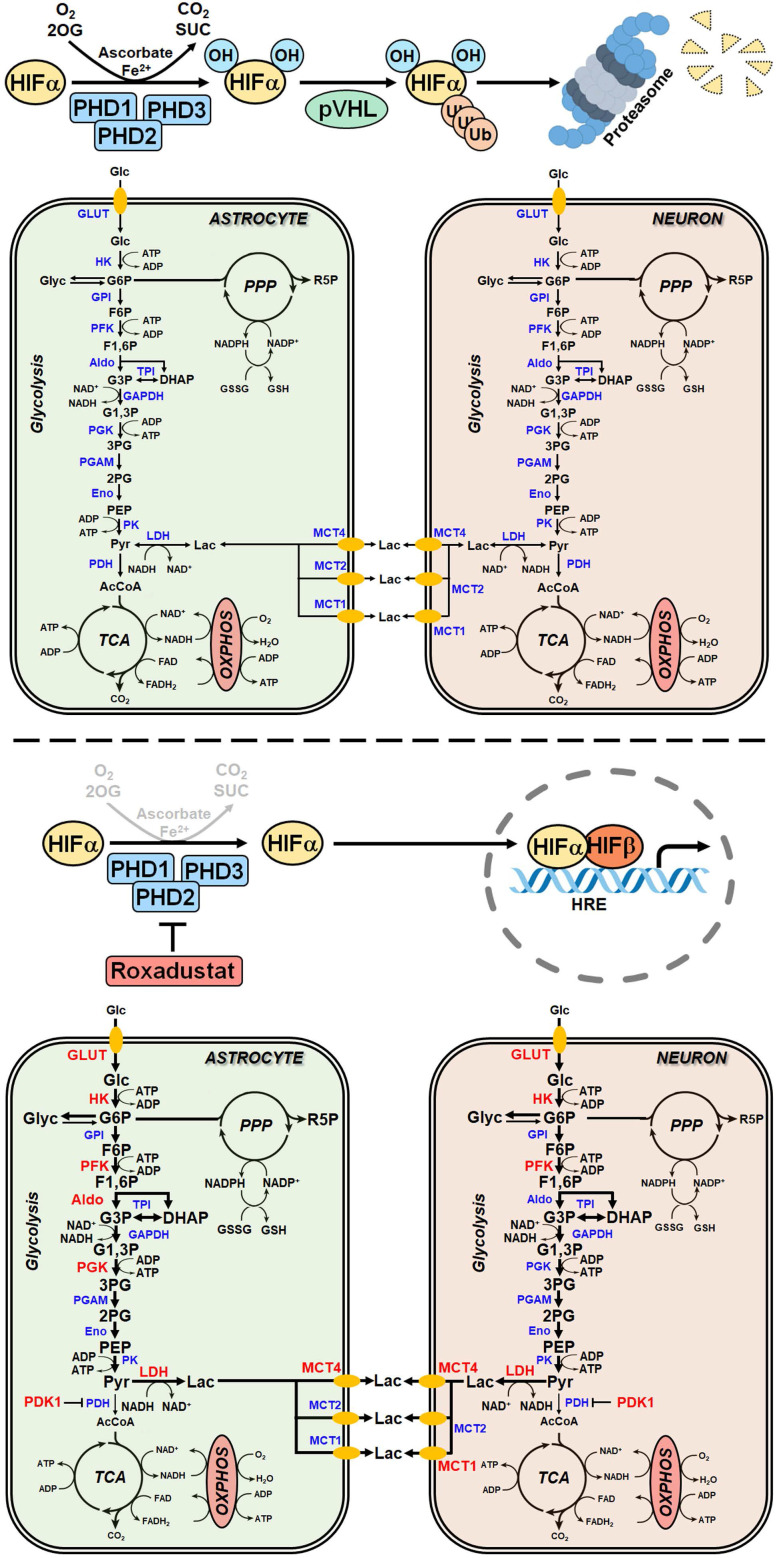
** Pharmacological activation of the HIF pathway improves the ischemic tolerance of brain-resident cells by reprogramming of glucose metabolism.** Preventing the destabilization and degradation of HIF-1α in neurons and astrocytes by pharmacological inhibition of PHDs using roxadustat increases their rate of glucose uptake, glycogenesis, glycolysis and the release of lactate into the extracellular space through HIF-1-dependent upregulation of GLUT1, key enzyme in glycogen synthesis, glycolytic enzymes and MCTs, respectively. Along this line, elevating cellular glucose stores concomitant with increasing the capacity for O_2_-independent glycolytic ATP production may represents an efficient metabolic adaptation of brain-resident cells to survive glucose and oxygen depletion during ischemic stroke. 2OG: 2-oxoglutarate; 2PG: 2-phosphoglycerate; 3PG: 3-phosphoglycerate; AcCoA: acetyl-coenzyme A; Aldo: fructose-bisphosphate aldolase; CO_2_: carbon dioxide; DHAP: dihydroxyacetonephosphate; Eno: enolase; F1,6P: fructose-1,6-bisphosphate; F6P: fructose-6-phosphate; G1,3P: 1,3-bisphosphoglycerate; G3P: glyceraldehyde-3-phosphate; G6P: glucose-6-phosphate; GAPDH: glyceraldehyde-3-phosphate dehydrogenase; Glc: glucose; GLUT: glucose transporter; Glyc: glycogen; GPI: glucose-6-phosphate isomerase; GSH: glutathione; HK: hexokinase; HIF: hypoxia-inducible factor; HRE: hypoxia response element; Lac: lactate; LDH: lactate dehydrogenase; MCT: monocarboxylate transporter; MS: mass spectrometry; NAD: nicotinamide adenine dinucleotide; NADP: nicotinamide adenine dinucleotide phosphate; O_2_: oxygen; OXPHOS: oxidative phosphorylation; PDH: pyruvate dehydrogenase; PDK1: pyruvate dehydrogenase kinase 1; PEP: phosphoenolpyruvate; PFK: phosphofructokinase; PGAM: phosphoglycerate mutase; PGK: phosphoglycerate kinase; PHD: prolyl-4-hydroxylase domain; PK: pyruvate kinase; PPP: pentose-phosphate pathway; Pyr: pyruvate; R5P: ribose-5-phosphate; SUC: succinate; TCA: tricarboxylic acid; TPI: triose-phosphate isomerase.

In summary, HIF-dependent cell metabolism reprogramming was thought for long to play a major role for the adaptation to hypoxia/ischemia, however the exact underlying mechanisms remain unresolved. Using genetic and pharmacological approaches to activate HIF in the murine brain *in vivo* and in primary neurons and astrocytes *in vitro* we prove that HIF-1-mediated metabolic reprogramming alleviates the intrinsic vulnerability of brain-resident cells to ischemic stress. We further underscore the physiological importance of the PHD2-HIF-axis by showing that HIF-1 promotes glycogen synthesis and initiates a metabolic shift to glycolytic energy production by increasing glucose uptake, glycolytic flux, pyruvate-to-lactate flux and lactate secretion in astrocytes and neurons (Figure 9) resulting in improved intrinsic tolerance to ischemia.

## Supplementary Material

Supplementary figures and tables.

## Abbreviations

aCSF: artificial cerebral spinal fluid
AldoA: fructose-bisphosphate aldolase A
ANLS: astrocyte-to-neuron lactate shuttle
APC/C: anaphase-promoting complex/cyclosome
ATP: adenosine triphosphate
BNIP3: BCL2/adenovirus E1B 19 kDa protein-interacting protein 3
BNIP3L: BNIP3-like
CAMK2a: calcium/calmodulin-dependent protein kinase II alpha
CBF: cortical blood flow
COX2: cyclooxygenase-2
COX4: cytochrome c oxidase subunit 4
DCA: dichloroacetate
DEC1: deleted in esophageal cancer 1
DIV: days *in vitro*
ECAR: extracellular acidification rate
ETC: electron transport chain
F2,6P2: fructose 2,6-bisphosphate
FIH: factor inhibiting HIF
G6P: glucose-6-phosphate
G6PD: glucose-6-phosphate dehydrogenase
GBE1: 1,4-alpha-glucan branching enzyme 1
GC: gas chromatography
GLUT1: glucose transporter 1
GSH: glutathione
GYS1: glycogen synthase 1
HK2: hexokinase 2
HIF: hypoxia-inducible factor
HRE: hypoxia response element
IL1β: interleukin-1 beta
I/R: ischemia/reperfusion
LC: liquid chromatography
LDH: lactate dehydrogenase
LonP1: lon protease 1
MCA: middle cerebral artery
MCP-1: monocyte chemoattractant protein-1
MCT: monocarboxylate transporter
MS: mass spectrometry
MSEA: metabolite set enrichment analysis
NAD: nicotinamide adenine dinucleotide
NADP: nicotinamide adenine dinucleotide phosphate
NRF2: NF-E2-related factor 2
O_2_: oxygen
OCR: oxygen consumption rate
OGD: oxygen-glucose deprivation
OHSC: organotypic hippocampal slice culture
OXPHOS: oxidative phosphorylation
PDHA1: pyruvate dehydrogenase E1 alpha subunit
PDK1: pyruvate dehydrogenase kinase 1
PFK1: phosphofructokinase 1
PFKFB3: 6-phosphofructo-2-kinase/fructose-2,6-bisphosphatase-3
PGC-1α: peroxisome proliferator-activated receptor gamma coactivator 1 alpha
PGK1: phosphoglycerate kinase 1
PGM1: phosphoglucomutase 1
PHD: prolyl-4-hydroxylase domain
PHI: PHD inhibitor
PPP: pentose-phosphate pathway
PYGB: glycogen phosphorylase B
ROS: reactive oxygen species
TCA: tricarboxylic acid
TNFα: tumor necrosis factor alpha
VEGF: vascular endothelial growth factor
