# Supplementary material for: Activation of the hypoxia-inducible factor pathway protects against acute ischemic stroke by reprogramming central carbon metabolism
Source: Theranostics. 2024 Apr 29;14(7):2856–80. doi: 10.7150/thno.88223 (PMC11103502; doi:10.7150/thno.88223)
Supplement: Supplementary file 1 — Supplementary figures and tables. [file thnov14p2856s1.pdf]

SUPPLEMENTARY MATERIAL

Activation of the hypoxia-inducible factor pathway protects against acute ischemic stroke by reprogramming central carbon metabolism

Sarah Madai[1], Pinar Kilic[1], Rolf M. Schmidt[2], Carlos Bas-Orth[2, *], Thomas Korff[1], Michael Büttner[3], Glynis Klinke[3], Gernot Poschet[3], Hugo H. Marti[1], Reiner Kunze[1]

[1] Institute of Physiology and Pathophysiology, Department of Cardiovascular Physiology, Heidelberg University, Heidelberg, Germany

[2] Department of Medical Cell Biology, Institute for Anatomy and Cell Biology, Heidelberg University, Heidelberg, Germany

[3] Metabolomics Core Technology Platform, Centre for Organismal Studies, Heidelberg University, Heidelberg, Germany

* present address: Institute of Clinical Neuroanatomy, Goethe University Frankfurt, Frankfurt, Germany

Address for correspondence:

Reiner Kunze
Heidelberg University
Institute of Physiology and Pathophysiology
Im Neuenheimer Feld 326
69120 Heidelberg, Germany
Phone +49 6221 54-4047
Fax +49 6221 54-8224
Email reiner.kunze@mail.de

Table S1 List of primers used to genotype mice.

Allele	Primer sequence (5´-3´)	Size (bp) of amplicon
Camk2a:cre	Fwd GGTTCTCCGTTTGCACTCAGGA	400
	Rev GCTTGCAGGTACAGGAGGTAGT	
Hif1a floxed	Fwd GCAGTTAAGAGCACTAGTTG	wt: 215
	Rev GGAGCTATCTCTCTAGACC	floxed: 250
Hif2a floxed	Fwd GAGAGCAGCTTCTCCTGGAA	wt: 182
	Rev TGTAGGCAAGGAAACCAAGG	floxed: 220
Phd2 floxed	Fwd CTCACTGACCTACGCCGTGT	wt: 220
	Rev CGCATCTTCCATCTCCATTT	floxed: 386

Table S2 Overview of animals that met defined exclusion criteria.

Animals that met the following criteria were excluded from end-point analyses: (1) death during surgery due to procedural or anesthetic problems, (2) death before sampling, (3) intracerebral hemorrhage, (4) structural brain abnormalities (e.g. hydrocephalus), and (5) no infarction.

mouse line/strain		number of mice met exclusion criteria (% of total animals)					
		(1)	(2)	(3)	(4)	(5)	total
Phd2$^{f/f}$		–	1	–	–	1	2 / 21 (10%)
nPhd2$^{\Delta/\Delta}$		–	–	1	1	–	2 / 22 (9%)
Phd2/Hif1a/Hif2a$^{fff/fff}$		–	3	–	–	–	3 / 23 (13%)
nPhd2/Hif1a/Hif2a$^{\Delta\Delta\Delta/\Delta\Delta\Delta}$		–	4	–	–	–	4 / 25 (16%)
C57BL/6	0 mg/kg Roxa	–	–	–	–	–	0 / 9 (0%)
	10 mg/kg Roxa	–	1	–	–	–	1 / 9 (11%)
C57BL/6	0 mg/kg Roxa	–	1	–	–	–	1 / 19 (5%)
	40 mg/kg Roxa	–	–	–	–	–	0 / 19 (0%)

Table S3 LC gradient scheme for the separation of energy carrier metabolites.

Time (min)	Flow (mL/min)	Phase A (%)	Phase B (%)
0	0.40	5	95
0.5	0.40	5	95
0.51	0.35	5	95
5	0.35	90	10
5.1	0.30	90	10
5.2	0.30	100	0
10	0.30	100	0
10.3	0.40	5	95
15	0.40	5	95

Table S4 Metabolite multiple-reaction monitoring in positive and negative ion mode. First transition is used as quantifier transition, the second as qualifier transition.

Compound ID	RT (min)	Q1	Q3	DP	EP	CE	CXP
ADP_1	4.86	426	79	-90	-10	-75	-12
ADP_2	4.86	426	134.064	-90	-10	-30	-15
AMP_1	4.53	346	78.9	-80	-10	-74	-11
AMP_2	4.53	346	134.039	-80	-10	-37	-15
ATP_1	5.23	506	158.9	-170	-10	-36	-19
ATP_2	5.23	506	408.027	-170	-10	-29	-35
GDP_1	5.2	442.1	150.1	-80	-10	-33	-9
GDP_2	5.2	442.1	344.065	-80	-10	-25	-34
GDP-Mannose_1	5.08	604.2	442	-140	-10	-36	-31
GDP-Mannose_2	5.08	604.2	424.005	-140	-10	-41	-18
GMP_1	4.85	362	78.9	-70	-10	-75	-10
GMP_2	4.85	362	211.028	-70	-10	-24	-17
GTP_1	5.61	522	159	-130	-10	-42	-8
GTP_3	5.61	522	424.044	-130	-10	-29	-17
UDP-GlcNAc_1	4.68	606.1	385	-150	-10	-37	-18
UDP-GlcNAc_2	4.53	606.1	78.928	-150	-10	-95	-11
UDP-Glucose-Galactose_1	4.82	565.1	323.1	-130	-10	-32	-29
UDP-Glucose-Galactose_2	4.68	565.1	78.924	-130	-10	-95	-20
Adenine_1	1.88	136.4	91.9	110	10	38	10
Adenine_2	1.88	136.4	93.947	110	10	27	10
Adenosine_1	2.076	267.9	136.1	80	10	20	17
Adenosine_2	2.076	267.9	119.06	80	10	61	7

ADPR_1	4.56	560.1	348.1	90	10	24	12
ADPR_2	4.56	560.1	136.083	90	10	37	12
Cytidine_1	2.891	244.1	112	10	10	22	13
Cytidine_2	2.891	244.1	94.967	10	10	57	14
Cytosine_1	2.37	112.4	68.9	90	10	23	8
Cytosine_2	2.37	112.4	67.89	90	10	32	8
FAD_1	4.33	786.2	348.1	120	10	31	16
FAD_2	4.33	786.2	439.153	120	10	42	21
Guanine_1	2.718	152.1	110	90	10	27	10
Guanine_2	2.718	152.1	107.004	90	10	34	13
Guanosine_1	3.08	284.1	152.1	60	10	17	12
Guanosine_2	3.06	284.1	135.055	60	10	52	7
Hypoxanthine_1	2.009	137	110	90	10	27	13
Hypoxanthine_2	2.009	137	93.979	90	10	27	11
Inosine_1	2.61	269.2	137.1	50	10	14	9
Inosine_2	2.61	269.2	110.049	50	10	56	16
NA_1	2.62	124.006	77.874	70	10	30	10
NA_2	2.62	124.006	79.912	70	10	27	35
NAD_1	4.72	663.924	427.9	6	10	35	22
NAD_2	4.72	663.924	524	6	10	25	22
NADH_1	4.37	665.953	513.9	60	10	33	26
NADH_2	4.37	665.953	301.9	60	10	47	16
NADP_1	5.22	743.915	603.9	66	10	29	30
NADP_2	5.22	743.915	507.9	66	10	39	24

NADPH_1	4.98	745.927	302	60	10	45	28
NADPH_2	4.98	745.927	108	60	10	113	12
NAMN_1	4.92	335.9	124.1	30	10	16	11
NAMN_2	4.92	335.9	96.94	30	10	45	16
Nicotinamide_1	1	123.1	79.9	70	10	28	9
Nicotinamide_2	1	123.1	77.888	70	10	31	8
N-Methylnicotinamide_1	0.86	137.1	79.9	70	10	29	10
N-Methylnicotinamide_2	0.86	137.1	108.05	70	10	23	15
NMN_1	5.1	335	123	30	10	19	14
NMN_2	5.1	335	96.959	30	10	43	10
NR_1	3.93	256.266	124.092	40	10	15	6
NR_2	3.93	256.266	107.058	40	10	41	11
SAH_1	4.37	385.104	136.098	70	10	22	13
SAH_2	4.37	385.104	250.131	70	10	18	35
SAM_1	6.06	400.296	299.113	70	10	18	27
SAM_2	6.06	400.296	136.098	70	10	18	27
Uracil_1	1.99	113.1	69.9	60	10	21	13
Uridine_1	1.99	245.1	113	50	10	14	12
NAAD_1	4.72	666.2	136.1	70	10	61	16
NAAD_2	4.72	666.2	543.074	70	10	21	18

ADP: Adenosine di-phosphate; ADPR: Adenine diphosphate ribose; AMP: Adenosine mono-phosphate; ATP: Adenosine tri-phosphate; FAD: Flavin adenine dinucleotide; GDP: Guanosine di-phosphate; GMP: Guanosine mono-phosphate; GTP: Guanosine tri-phosphate; NA: Nicotinic acid; NAAD: Nicotinic acid adenine dinucleotide; NAD: Nicotinamide adenine dinucleotide; NADP: Nicotinamide adenine dinucleotide phosphate; NAM: Nicotinamide; NAMN: Nicotinic acid mononucleotide; NMN: Nicotinamide mononucleotide; NR: Nicotinamide riboside; SAH: S-Adenosylhomocystein; SAM: S-Adenosylmethionin; UDP: Uridine di-phosphate

Table S5 List of antibodies used for immunoblotting.

Antibody type	Immunogen	Host species	Clonality	Conjugate	Supplier	Catalog no.
Primary	hu beta Tubulin	rb	polyclonal	/	Abcam	ab6046
Primary	hu HIF-1α	rb	polyclonal	/	Novus	NB100-449
Primary	hu PDK1	rb	polyconal	/	Enzo Life Sciences	ADI-KAP-PK112
Primary	hu phospho-PDHA1	rb	polyclonal	/	Merck Millipore	ABS204
Primary	hu PDHA1	ms	monoclonal	/	Thermo Fisher	45-6600
Primary	hu PFKFB3	rb	monoclonal	/	Cell Signaling	13123
Primary	ms PHD2	rb	polyclonal	/	Novus	NB100-2219
Primary	hu TBP	ms	monoclonal	/	Abcam	ab818
Secondary	rb IgG	gt	polyclonal	HRP	Thermo Fisher	31460
Secondary	ms IgG	gt	polyclonal	HRP	Thermo Fisher	31432

gt: goat; HIF, hypoxia-inducible factor; HRP: horseradish peroxidase; hu: human; ms: mouse; PDK1: pyruvate dehydrogenase kinase 1; PDHA1: pyruvate dehydrogenase E1 component subunit alpha; PFKFB3: 6-Phosphofructo-2-Kinase/Fructose-2,6-Biphosphatase 3; PHD2: prolyl-4-hydroxylase domain 2; rb: rabbit; TBP: TATA-box-binding protein

Table S6 List of primers used for quantitative real-time RT-PCR.

Gene	Forward primer sequence (5´-3´)	Reverse primer sequence (5´-3´)
Aldoa	CCATGCTTGCACCCAGAAAT	AGACAGGAAAGTGACCCCAG
Bnip3	GCTCCCAGACACCACAAGAT	TGAGAGTAGCTGTGCGCTTC
Bnip3l	TGAGGAAGAGTGGAGCCATG	TATAGATGCCGAGCCCCAAG
Cox2	AGAAGGAAATGGCTGCAGAA	GCTCGGCTTCCAGTATTGAG
Cox4i2	CATTGGATTCACGGCTCTGG	TGCTCTTCATGTCCAGGAGG
Dec1	TATCTCATCCCACCATCGGC	GCCAAAGGAGAAGGGAGTCT
Gbe1	AGGATGTATCAGGGATGCCG	CAAGGTAGCGTCGATTGGTG
Glut1	GCTGTGCTTATGGGCTTCTC	AGAGGCCACAAGTCTGCATT
Glut3	TGTCACAGGAGAAGCAGGTG	GCTCCAATCGTGGCATAGAT
Gys1	CTGGCCGCTATGAGTTTTCC	TGGCTGTGTCCCATAGTTGT
Hif1a	TGCTCATCAGTTGCCACTTC	CGGCATCCAGAAGTTTTCTC
Hk2	TACCACACACCCTACAGCAG	CTCGGGAATGGCGTAGATCT
Il1b	ATAACCTGCTGGTGTGTGACG	GGTGGAGAGCTTTCAGCTCAT
Il6	AGTTGCCTTCTTGGGACTGA	TCCACGATTTCCCAGAGAAC
Ldha	CCGTTACCTGATGGGAGAGA	GTAGGCACTGTCCACCACCT
Lonp1	AACTACCTGGACTGGCTGAC	ACGCTTCTTCACATCCTCCA
Mcp1	CCCAATGAGTAGGCTGGAGA	TCTGGACCCATTCCTTCTTG
Mct1	TGGTGGTTGTCTGTCTGGTT	TTGAAAGCAAGCCCAAGACC
Mct2	GTTTGCTCGGCCTTCTGTAG	ACGTAGACCACCAAAGCTGA
Mct4	TCAATCATGGTGCTGGGACT	AGGATGGTGAGAAGGAAGGC
Pdk1	GGCGGCTTTGTGATTTGTAT	ACCTGAATCGGGGGATAAAC
Pfk1	ATGGCAAAGCTATCGGTGTC	ACACAGTCCCATTTGGCTTC
Pfkfb3	TGACTCGCTACCTCAACTGG	AAGGCACACTGTTTTCGGAC
Pgk1	CAAGGCTTTGGAGAGTCCAG	TGTGCCAATCTCCATGTTGT
Pgm1	GCATTCCGTACTTCCAGCAG	CCAAAGCTCTCCTCTCCACA

Phd1	GCTAGGCTGAGGGAGGAAGT	CCCCAAGAAGTTGTCCTTGA
Phd2	TCACGCATCTTCCATCTCCA	GGCAACGGAACAGGCTATG
Phd3	GCTATCCAGGAAATGGGACA	TGGCGTCCCAATTCTTATTC
Ppargc1a	TCAGAACCATGCAGCAAACC	TTGGTGTGAGGAGGGTCATC
Pygb	GGGATAAGGCCTGGGAGATC	TCCAAGTGCCTCTGGTTGAT
Rps12	GAAGCTGCCAAAGCCTTAGA	AACTGCAACCAACCACCTTC
Tnf	CGTCAGCCGATTTGCTATCT	CGGACTCCGCAAAGTCTAAG
Vegfa	GTACCTCCATGCCAAGT	ACTCCAGGGCTTCATCGTTA

Aldoa: fructose-bisphosphate aldolase A; *Bnip3*: BCL2/adenovirus E1B 19kDa interacting protein 3; *Bnip3l*: Bnip3-like; *Cox2*: cyclooxygenase-2; *Cox4i2*: cytochrome c oxidase subunit 4 isoform 2; *Dec1*: deleted in esophageal cancer 1; *Gbe1*: 1,4-alpha-glucan branching enzyme 1; *Glut*: glucose transporter; *Gys1*: glycogen synthase 1; *Hif*: hypoxia-inducible factor; *Hk2*: hexokinase 2; *Il1b*: interleukin-1 beta; *Ldh*: lactate dehydrogenase; *Lonp1*: lon protease 1; *Mcp-1*: monocyte chemoattractant protein-1; *Mct*: monocarboxylate transporter; *Pdk1*: pyruvate dehydrogenase kinase 1; *Pfk1*: phosphofructokinase 1; *Pfkfb3*: 6-phosphofructo-2-kinase/fructose-2,6-bisphosphatase 3; *Pgk1*: phosphoglycerate kinase 1; *Pgm1*: phosphoglucomutase 1; *Phd*: prolyl-4-hydroxylase domain; *Ppargc1a*: peroxisome proliferator-activated receptor gamma co-activator 1 alpha; *Pygb*: glycogen phosphorylase B; *Rps12*: 40S ribosomal protein S12; *Tnf*: tumor necrosis factor alpha; *Vegfa*: vascular endothelial growth factor A

Table S7 Results of semi-targeted metabolomics.

compound name	database ID			mean ± SD		*p*-value
	HMDB	PubChem	KEGG	*Phd2 $^{flox/flox}$*	*nPhd2 $^{\Delta/\Delta}$*	
L-Serine	HMDB0000187	5951	C00065	1.60E+07 ± 3.62E+06	2.56E+07 ± 2.38E+06	0.0011
Pyruvic acid	HMDB0000243	1060	C00022	7.88E+06 ± 1.16E+06	5.35E+06 ± 7.04E+05	0.0031
L-Proline	HMDB0000162	145742	C00148	2.96E+06 ± 8.80E+05	4.63E+06 ± 2.56E+05	0.0037
L-Glutamic acid	HMDB0000148	33032	C00302	4.64E+07 ± 1.72E+06	5.54E+07 ± 5.01E+06	0.0052
N-Acetyl-L-glutamic acid	HMDB0001138	70914	C00624	1.12E+07 ± 6.53E+06	2.33E+07 ± 4.22E+06	0.0082
D-Sorbitol	HMDB0000247	5780	C00794	1.55E+07 ± 2.99E+06	2.22E+07 ± 3.45E+06	0.0115
Palmitic Acid	HMDB0000220	985	C00249	1.30E+07 ± 9.51E+06	4.82E+07 ± 2.30E+07	0.0133
Fumaric acid	HMDB0000134	444972	C00122	1.66E+0 7 ± 2.11E+06	2.02E+07 ± 1.70E+06	0.0173
D-Fructose	HMDB0000660	439709	C00095	4.27E+06 ± 9.10E+05	7.80E+06 ± 2.70E+06	0.0245
Butanoic acid	HMDB0000039	264	C00246	2.26E+06 ± 4.67E+05	3.12E+06 ± 4.41E+05	0.0262
Glyceric acid	HMDB0000139	439194	C00258	7.46E+06 ± 6.94E+05	9.20E+06 ± 1.29E+06	0.0286
L-Valine	HMDB0000883	6287	C00183	1.55E+07 ± 1.23E+06	1.82E+07 ± 1.86E+06	0.0299
Glycerol-2-phosphate	HMDB0002520	2526	C02979	8.52E+05 ± 5.48E+04	1.06E+06 ± 1.67E+05	0.0302
Lactic acid	HMDB0000190	107689	C00186	4.30E+07 ± 4.16E+06	4.88E+07 ± 3.22E+06	0.0385
Phosphoric acid	HMDB0001429	1004	C00009	1.43E+07 ± 2.60E+06	1.83E+07 ± 2.54E+06	0.0393
Glycerol	HMDB0000131	753	C00116	2.74E+06 ± 3.53E+05	2.28E+06 ± 2.31E+05	0.0413
L-Phenylalanine	HMDB0000159	6140	C00079	1.54E+06 ± 8.96E+05	3.66E+06 ± 1.09E+06	0.0417
5-Hydroxymethyl-2-furoic acid	HMDB0002432	80642	C20448	7.76E+05 ± 4.61E+05	1.77E+06 ± 5.63E+05	0.0431
Alpha-Ketoglutaric acid	HMDB0000208	51	C00026	7.55E+05 ± 7.03E+04	8.67E+05 ± 7.97E+04	0.0461

Pyroglutamic acid	HMDB0000267	7405	C01879	1.14E+08 ± 1.56E+07	9.92E+07 ± 3.48E+06	0.0684
Scyllo-Inositol	HMDB0006088	892	C06153	5.04E+07 ± 5.54E+06	4.49E+07 ± 3.17E+06	0.0916
Pyrazine	HMDB0034176	9061	C02018	1.65E+06 ± 5.12E+04	1.85E+06 ± 2.47E+05	0.1056
2-hydroxymalonic acid	HMDB0035227	45	C02287	4.10E+06 ± 2.81E+06	7.14E+06 ± 2.67E+06	0.1178
L-Aspartic acid	HMDB0000191	5960	C00049	2.60E+08 ± 4.82E+07	2.18E+08 ± 2.74E+07	0.1296
Urea	HMDB0000294	1176	C00086	1.55E+08 ± 3.85E+07	2.05E+08 ± 5.67E+07	0.1409
N-Acetyl-D-glucosamine	HMDB0000215	439174	C00140	1.40E+06 ± 1.72E+05	9.60E+05 ± 6.21E+05	0.1664
Cholesterol	HMDB0000067	5997	C00187	2.02E+07 ± 2.04E+07	4.57E+07 ± 3.30E+07	0.1796
3-Hydroxy-3-methylglutaric acid	HMDB0000355	1662	C03761	8.00E+06 ± 1.86E+06	9.48E+06 ± 1.47E+06	0.2012
3-Hydroxypropionic acid	HMDB0000700	68152	C01013	4.54E+06 ± 1.10E+06	5.67E+06 ± 1.40E+06	0.2150
Oxalic acid	HMDB0002329	971	C00209	3.74E+07 ± 8.46E+06	4.40E+07 ± 7.08E+06	0.2202
3,4-Dihydroxyphenylacetic acid	HMDB0001336	547	C01161	1.12E+05 ± 4.84E+04	1.45E+05 ± 3.08E+04	0.2375
D-Gluconic acid	HMDB0000625	10690	C00257	5.07E+05 ± 4.49E+05	8.68E+05 ± 5.55E+05	0.2910
Myo-Inositol	HMDB0000211	–	C00137	2.88E+08 ± 3.48E+07	2.65E+08 ± 2.85E+07	0.2963
D-Arabino-hexos-2-ulose	HMDB0029932	159630	–	8.42E+06 ± 3.23E+06	1.11E+07 ± 4.21E+06	0.2983
Ethylene glycol	HMDB0037790	174	C01380	1.05E+07 ± 3.22E+06	8.70E+06 ± 1.79E+06	0.3153
Acetic acid hydrazide	HMDB0060428	14039	C07447	2.70E+06 ± 7.73E+05	2.14E+06 ± 5.36E+05	0.3158
Ascorbic acid	HMDB0000044	54670067	C00072	1.30E+08 ± 2.94E+07	1.11E+08 ± 2.57E+07	0.3169
Erythritol	HMDB0002994	222285	C00503	2.51E+06 ± 9.10E+05	1.78E+06 ± 1.04E+06	0.3313
3-Hydroxybutyric acid	HMDB0000011	92135	C01089	1.18E+07 ± 2.82E+06	3.71E+07 ± 5.87E+07	0.3639
Caproic acid	HMDB0000535	8892	C01585	5.88E+05 ± 2.89E+05	4.35E+05 ± 2.11E+05	0.3649
L-Methionine	HMDB0000696	6137	C00073	5.53E+06 ± 9.26E+05	5.99E+06 ± 6.59E+05	0.3886

L-Threonine	HMDB0000167	6288	C00188	5.87E+07 ± 8.04E+06	6.41E+07 ± 1.08E+07	0.4020
2-Pentenoic acid	HMDB0032459	638122	–	6.77E+06 ± 2.87E+05	7.08E+06 ± 6.29E+05	0.4141
Malic acid	HMDB0000156	222656	C00149	4.20E+07 ± 3.79E+06	4.44E+07 ± 4.89E+06	0.4165
Ribitol	HMDB0000508	–	C00474	9.44E+06 ± 2.59E+06	8.34E+06 ± 1.65E+06	0.4480
L-Homoserine	HMDB0000719	12647	C00263	3.07E+06 ± 2.03E+06	2.29E+06 ± 9.43E+05	0.4584
Niacinamide	HMDB0001406	936	C00153	1.15E+07 ± 6.38E+06	8.29E+06 ± 6.76E+06	0.4682
Picolinic acid	HMDB0002243	1018	C10164	1.20E+06 ± 5.55E+05	9.40E+05 ± 8.46E+04	0.4726
Creatinine	HMDB0000562	588	C00791	1.60E+08 ± 3.35E+07	1.40E+08 ± 4.60E+07	0.4870
L-Alanine	HMDB0000161	5950	C00041	1.47E+08 ± 2.08E+07	1.38E+08 ± 1.92E+07	0.4979
D-Pinitol	HMDB0034219	–	C03844	1.10E+06 ± 7.39E+05	1.35E+06 ± 5.31E+05	0.5421
Aminomalonic acid	HMDB0001147	100714	C00872	1.73E+07 ± 6.46E+06	1.52E+07 ± 3.49E+06	0.5554
Alpha-Hydroxyglutaric acid	HMDB0059655	43	C02630	5.87E+07 ± 5.39E+06	6.06E+07 ± 4.37E+06	0.5563
Succinic acid	HMDB0000254	1738118	C00042	4.72E+07 ± 6.17E+06	4.51E+07 ± 5.12E+06	0.5699
L-Leucine	HMDB0000687	6106	C00123	1.63E+07 ± 1.11E+06	1.67E+07 ± 1.21E+06	0.5850
Glycine	HMDB0000123	750	C00037	3.64E+07 ± 6.64E+06	3.92E+07 ± 8.85E+06	0.5921
4-aminobutanoic acid	HMDB0000112	119	C00334	1.30E+08 ± 7.32E+06	1.27E+08 ± 8.95E+06	0.5986
Ribonic acid	HMDB0000867	5460677	C01685	2.15E+07 ± 2.71E+06	2.26E+07 ± 4.32E+06	0.6194
Ethanolamine	HMDB0000149	700	C00189	4.25E+07 ± 6.63E+06	4.08E+07 ± 4.03E+06	0.6219
Erythronic acid	HMDB0000613	2781043	–	3.57E+07 ± 4.32E+06	3.46E+07 ± 1.46E+06	0.6219
Phosphorylethanolamine	HMDB0000224	1015	C00346	5.82E+07 ± 9.55E+06	6.03E+07 ± 6.50E+06	0.6972
cis-Aconitic acid	HMDB0000072	643757	C00417	1.10E+06 ± 5.54E+05	9.99E+05 ± 4.79E+05	0.7665
D-Glucose	HMDB0000122	64589	C00031	2.96E+07 ± 9.53E+06	3.23E+07 ± 1.68E+07	0.7672

3-Phosphoglyceric acid	HMDB0000807	724	C00597	7.28E+06 ± 1.75E+06	6.92E+06 ± 1.99E+06	0.7874
Beta-Alanine	HMDB0000056	239	C00099	2.52E+07 ± 1.64E+07	2.72E+07 ± 8.87E+06	0.8177
Glycolic acid	HMDB0000115	757	C03547	1.38E+07 ± 1.51E+06	1.35E+07 ± 1.74E+06	0.8281
Glycerol-3-phosphate	HMDB0000126	439162	C00093	1.02E+08 ± 5.54E+07	9.39E+07 ± 5.18E+07	0.8312
Pantothenic acid	HMDB0000210	6613	C00864	3.70E+06 ± 1.02E+06	3.52E+06 ± 1.49E+06	0.8414
L-Isoleucine	HMDB0000172	6306	C00407	7.57E+06 ± 1.76E+06	7.67E+06 ± 8.65E+05	0.9127
Citric acid	HMDB0000094	311	C00158	6.76E+07 ± 1.27E+07	6.70E+07 ± 7.25E+06	0.9223
Uracil	HMDB0000300	1174	C00106	2.26E+06 ± 7.42E+05	2.29E+06 ± 2.66E+05	0.9228
Erythrono-1,4-lactone	HMDB0000349	5325915	–	1.17E+07 ± 1.26E+06	1.17E+07 ± 1.82E+06	0.9727
1,5-Anhydroglucitol	HMDB0002712	64960	C07326	1.76E+07 ± 4.55E+06	1.75E+07 ± 3.20E+06	0.9814

Table S8 Results from Quantitative Enrichment analysis.

**Pathway name**	**Raw p**	**Holm p**	**FDR**
Arginine and proline metabolism	0.0006	0.0160	0.0055
Glycine, serine and threonine metabolism	0.0008	0.0213	0.0055
Glyoxylate and dicarboxylate metabolism	0.0008	0.0213	0.0055
Cysteine and methionine metabolism	0.0010	0.0248	0.0055
D-Amino acid metabolism	0.0011	0.0282	0.0055
Sphingolipid metabolism	0.0011	0.0282	0.0055
Lipoic acid metabolism	0.0030	0.0701	0.0079
Glycolysis / Gluconeogenesis	0.0031	0.0701	0.0079
Alanine, aspartate and glutamate metabolism	0.0033	0.0701	0.0079
Citrate cycle (TCA cycle)	0.0033	0.0701	0.0079
Tyrosine metabolism	0.0033	0.0701	0.0079
Pyruvate metabolism	0.0033	0.0701	0.0079
Arginine biosynthesis	0.0052	0.0878	0.0115
Galactose metabolism	0.0100	0.1600	0.0176
Fructose and mannose metabolism	0.0101	0.1600	0.0176
Phenylalanine metabolism	0.0105	0.1600	0.0176
Phenylalanine, tyrosine and tryptophan biosynthesis	0.0105	0.1600	0.0176
Butanoate metabolism	0.0114	0.1600	0.0176
Fatty acid biosynthesis	0.0133	0.1600	0.0176
Fatty acid elongation	0.0133	0.1600	0.0176
Fatty acid degradation	0.0133	0.1600	0.0176
Biosynthesis of unsaturated fatty acids	0.0133	0.1600	0.0176
Starch and sucrose metabolism	0.0245	0.1720	0.0296
Amino sugar and nucleotide sugar metabolism	0.0245	0.1720	0.0296
Glycerolipid metabolism	0.0264	0.1720	0.0299
Pentose phosphate pathway	0.0286	0.1720	0.0299
Valine, leucine and isoleucine degradation	0.0299	0.1720	0.0299
Valine, leucine and isoleucine biosynthesis	0.0299	0.1720	0.0299
Pantothenate and CoA biosynthesis	0.0299	0.1720	0.0299

Table S9 Results of semi-targeted metabolomics.

compound name	database ID			mean ± SD		*p*-value
	HMDB	PubChem	KEGG	vehicle	Roxadustat	
Alpha-Ketoglutaric acid	HMDB0000208	51	C00026	9.20E+05 ± 5.59E+04	1.27E+06 ± 9.03E+04	0.0010
Alpha-D-Glucose	HMDB0003345	79025	C00267	8.63E+07 ± 1.12E+07	1.16E+08 ± 9.69E+06	0.0021
D-myo-inositol 3-phosphate	HMDB0000213	–	C01177	5.63E+07 ± 2.46E+07	1.08E+08 ± 1.61E+07	0.0042
Erythrono-1,4-lactone	HMDB0000349	5325915	–	2.69E+07 ± 4.34E+06	3.42E+07 ± 2.24E+06	0.0100
Citric acid	HMDB0000094	311	C00158	8.95E+07 ± 2.77E+07	1.63E+08 ± 5.22E+06	0.0108
Butanoic acid	HMDB0000039	264	C00246	1.18E+07 ± 6.19E+06	2.44E+07 ± 5.67E+06	0.0151
Creatinine	HMDB0000562	588	C00791	2.38E+08 ± 6.00E+06	2.68E+08 ± 1.78E+07	0.0152
Glycerol-2-phosphate	HMDB0002520	2526	C02979	9.51E+06 ± 3.39E+06	1.54E+07 ± 2.61E+06	0.0152
L-Glutamic acid	HMDB0000148	33032	C00025	2.31E+08 ± 8.51E+07	4.02E+08 ± 9.40E+07	0.0167
L-Threonine	HMDB0000167	6288	C00188	6.04E+07 ± 2.58E+07	9.14E+07 ± 5.27E+06	0.0299
2-Pentenoic acid	HMDB0032459	638122	–	1.28E+07 ± 6.14E+06	2.47E+07 ± 5.56E+06	0.0305
1,5-Anhydroglucitol	HMDB0002712	64960	C07326	3.67E+07 ± 2.35E+07	6.55E+07 ± 7.84E+06	0.0322
2-Phosphoglyceric acid	HMDB0000362	59	–	4.45E+05 ± 2.49E+05	2.12E+06 ± 1.43E+06	0.0325
Alpha-Hydroxyglutaric acid	–	163312007	C20267	6.13E+07 ± 1.28E+07	7.89E+07 ± 9.86E+06	0.0409
Lactic Acid	HMDB0000190	61503	C00186	3.45E+07 ± 2.25E+07	1.70E+08 ± 1.28E+08	0.0473
Picolinic acid	HMDB0002243	1018	C10164	1.79E+06 ± 3.53E+05	2.23E+06 ± 2.32E+05	0.0481
Fumaric acid	HMDB0000134	444972	C00122	1.89E+07 ± 8.71E+06	2.98E+07 ± 5.84E+06	0.0485
N-Acetylgalactosamine	HMDB0000212	84265	C01074	3.01E+07 ± 1.53E+07	4.67E+07 ± 5.04E+06	0.0492
Glyceric acid	HMDB0000139	439194	C00258	2.02E+07 ± 6.21E+06	2.87E+07 ± 5.32E+06	0.0492

5'-Methylthioadenosine	HMDB0001173	439176	C00170	4.80E+06 ± 2.26E+06	7.51E+06 ± 3.55E+05	0.0520
Adenosine	HMDB0000050	60961	C00212	1.17E+07 ± 8.70E+06	2.16E+07 ± 4.85E+06	0.0581
L-Serine	HMDB0000187	5951	C00065	2.31E+08 ± 7.04E+07	3.10E+08 ± 2.50E+06	0.0613
Benzoic Acid	HMDB0001870	243	C00539	1.49E+06 ± 6.13E+05	2.14E+06 ± 2.81E+05	0.0636
Pentanedioic acid	HMDB0000661	743	C00489	4.63E+06 ± 2.81E+06	1.77E+07 ± 1.38E+07	0.0726
Uridine	HMDB0000296	6029	C00299	3.45E+07 ± 6.50E+06	4.13E+07 ± 3.58E+06	0.0738
L-Leucine	HMDB0000687	6106	C00123	3.99E+07 ± 1.03E+07	5.09E+07 ± 6.53E+06	0.0776
2-Aminoadipic acid	HMDB0000510	469	C00956	2.67E+06 ± 1.29E+06	4.22E+06 ± 1.01E+06	0.0821
Oxalic acid	HMDB0002329	971	C00209	1.77E+07 ± 4.18E+06	2.58E+07 ± 8.30E+06	0.0869
Pipecolic acid	HMDB0000070	849	C00408	1.62E+08 ± 6.48E+07	8.02E+07 ± 3.47E+07	0.0954
Glycine	HMDB0000123	750	C00037	1.32E+07 ± 6.59E+06	2.04E+07 ± 4.82E+06	0.1200
Phosphorylethanolamine	HMDB0000224	1015	C00346	1.52E+08 ± 8.75E+07	2.16E+08 ± 2.74E+07	0.1595
Niacinamide	HMDB0001406	936	C00153	2.49E+07 ± 9.59E+06	3.55E+07 ± 7.83E+06	0.1785
Adenine	HMDB0000034	190	C00147	9.52E+06 ± 9.15E+06	1.56E+07 ± 1.88E+06	0.1844
Phosphoric acid	HMDB0002142	57424078	C00009	4.09E+07 ± 1.59E+07	5.15E+07 ± 4.61E+06	0.1902
2-Pyrrolidinone	HMDB0002039	12025	C11118	3.97E+07 ± 1.70E+06	4.31E+07 ± 3.74E+06	0.1964
L-Aspartic acid	HMDB0000191	5960	C00049	1.18E+08 ± 6.15E+07	6.78E+07 ± 4.95E+07	0.2144
4'-Hydroxyacetophenone	METPA0982	–	C10700	3.55E+06 ± 3.01E+06	1.80E+06 ± 1.52E+05	0.2178
Ribonic acid	HMDB0000867	5460677	C01685	6.09E+07 ± 2.30E+07	3.17E+07 ± 4.46E+07	0.2294
1-Monopalmitin	HMDB0011564	3084463	–	2.03E+06 ± 8.51E+05	2.82E+06 ± 9.47E+05	0.2333
2-Aminomalonic acid	HMDB0001147	100714	C00872	3.75E+07 ± 1.58E+07	4.78E+07 ± 8.56E+06	0.2382
Glycerol-3-phosphate	HMDB0000126	439162	C00093	6.70E+07 ± 8.85E+07	1.44E+07 ± 2.84E+07	0.2414

Pyroglutamic acid	HMDB0000267	7405	C01879	1.84E+08 ± 1.20E+08	4.02E+08 ± 2.63E+08	0.2465
D-Fructose 1,6-bisphosphate	HMDB0001058	445557	C05378	3.09E+06 ± 1.49E+06	9.99E+06 ± 1.10E+07	0.2594
2-Propenoic acid	HMDB0031647	6581	C19501	4.59E+06 ± 2.50E+06	1.06E+07 ± 1.05E+07	0.3056
3-Phosphoglyceric acid	HMDB0000807	724	C00597	4.49E+07 ± 4.08E+07	7.24E+07 ± 7.07E+06	0.3134
L-Phenylalanine	HMDB0000159	6140	C00079	3.18E+07 ± 2.64E+07	5.06E+07 ± 3.07E+07	0.3292
Malic acid	HMDB0000744	525	C03668	1.27E+08 ± 3.43E+07	1.56E+08 ± 5.27E+07	0.3433
Uracil	HMDB0000300	1174	C00106	1.38E+07 ± 1.75E+06	1.47E+07 ± 1.07E+06	0.3561
L-Alanine	HMDB0000161	5950	C00041	5.03E+07 ± 2.37E+07	6.11E+07 ± 8.27E+06	0.3652
Beta-D-Glucose 6-phosphate	HMDB0003498	439427	C01172	1.51E+07 ± 4.51E+06	1.23E+07 ± 4.70E+06	0.3674
Pyruvic acid	HMDB0000243	1060	C00022	2.69E+06 ± 1.16E+06	2.12E+06 ± 6.98E+05	0.3748
Propanoic acid	HMDB0000237	1032	C00163	6.12E+08 ± 8.33E+07	4.61E+08 ± 2.99E+08	0.4404
Homoserine	HMDB0000719	12647	C00263	2.65E+07 ± 4.73E+07	4.21E+06 ± 7.02E+05	0.4596
Tromethamine	HMDB0240288	6503	C07182	5.00E+06 ± 3.56E+06	6.75E+06 ± 3.26E+06	0.4737
Erythronic acid	HMDB0000613	2781043	–	1.01E+08 ± 1.63E+07	9.46E+07 ± 1.65E+07	0.5694
Glycolic acid	HMDB0000115	757	C03547	1.69E+07 ± 1.53E+07	1.22E+07 ± 1.01E+07	0.5823
Malonic acid	HMDB0000691	867	C04025	9.07E+05 ± 5.97E+05	1.07E+06 ± 2.95E+05	0.6407
Tyrosine	HMDB0000158	6057	C00082	2.91E+07 ± 3.44E+07	2.21E+07 ± 2.85E+06	0.6618
L-Valine	HMDB0000883	6287	C00183	5.53E+07 ± 1.96E+07	5.95E+07 ± 1.07E+07	0.6885
Hexanoic acid	HMDB0000535	8892	C01585	1.79E+07 ± 2.11E+07	1.31E+07 ± 1.42E+07	0.6886
Succinic acid	HMDB0000254	1110	C00042	2.27E+08 ± 2.07E+07	2.38E+08 ± 5.67E+07	0.6960
Pantothenic acid	HMDB0000210	6613	C00864	2.10E+07 ± 9.97E+06	2.25E+07 ± 2.00E+06	0.7580
Pyrazine	HMDB0034176	9261	C02018	6.58E+07 ± 3.92E+07	7.15E+07 ± 2.58E+07	0.7919

D-Mannitol	HMDB0000765	6251	C00392	5.39E+07 ± 3.92E+07	6.02E+07 ± 3.39E+07	0.7937
Beta-Alanine	HMDB0000056	239	C00099	6.53E+07 ± 2.78E+07	6.07E+07 ± 3.51E+07	0.8259
D-Gluconic acid	HMDB0000625	10690	C00257	1.72E+06 ± 1.37E+06	1.83E+06 ± 8.46E+05	0.8875
4-Hydroxybutanoic acid	HMDB0000549	7302	C01770	4.33E+06 ± 2.92E+06	4.58E+06 ± 2.71E+06	0.8954
Uridine-5'-monophosphate	HMDB0000288	6030	C00105	1.80E+06 ± 4.32E+05	1.84E+06 ± 4.14E+05	0.9017
Homovanillic Acid	HMDB0000118	1738	C05582	1.14E+06 ± 7.52E+05	1.18E+06 ± 1.13E+05	0.9047
L-Isoleucine	HMDB0000172	6306	C00407	2.18E+07 ± 5.48E+06	2.22E+07 ± 4.58E+06	0.9073
2-keto-D-gluconic acid	METPA0825	–	C06473	1.17E+07 ± 6.30E+06	1.20E+07 ± 5.15E+06	0.9214
3-Hydroxyisovaleric acid	HMDB0000754	69362	C20827	2.71E+06 ± 1.91E+06	2.84E+06 ± 1.59E+06	0.9297
Tacrine	HMDB0014526	1935	C01453	7.77E+06 ± 6.41E+06	7.45E+06 ± 7.62E+05	0.9372
D-(-)-Fructose	HMDB0000660	439709	C02336	1.03E+07 ± 4.72E+06	1.05E+07 ± 2.87E+06	0.9469
2-Hydroxy-3-methylbutyric acid	HMDB0000407	99823	–	9.18E+06 ± 2.03E+06	9.12E+06 ± 2.08E+06	0.9648
Phenylalanine	HMDB0000159	6140	C00079	2.01E+07 ± 8.06E+06	1.99E+07 ± 5.43E+06	0.9679
3-Hydroxybutyric acid	HMDB0000357	441	C01089	3.04E+07 ± 1.76E+07	3.03E+07 ± 3.65E+06	0.9866
D-Fructose	HMDB0000660	439709	C02336	2.32E+07 ± 2.48E+07	2.31E+07 ± 1.92E+07	0.9923
Ascorbic acid	HMDB0000044	54670067	C01041	4.83E+07 ± 5.81E+07	4.83E+07 ± 3.90E+07	0.9997

Table S10 Results from Quantitative Enrichment analysis.

Pathway name	Raw p	Holm p	FDR
Alanine, aspartate and glutamate metabolism	0.00008	0.0012	0.0005
Citrate cycle (TCA cycle)	0.00008	0.0012	0.0005
Glyoxylate and dicarboxylate metabolism	0.00009	0.0013	0.0005
Lipoic acid metabolism	0.0003	0.0039	0.0012
Amino sugar and nucleotide sugar metabolism	0.0010	0.0118	0.0031
Glycolysis / Gluconeogenesis	0.0021	0.0232	0.0042
Fructose and mannose metabolism	0.0021	0.0232	0.0042
Galactose metabolism	0.0021	0.0232	0.0042
Butanoate metabolism	0.0132	0.1060	0.0235
Glycine, serine and threonine metabolism	0.0263	0.1840	0.0421
Valine, leucine and isoleucine biosynthesis	0.0299	0.1840	0.0435
Arginine biosynthesis	0.0485	0.2420	0.0492
Tyrosine metabolism	0.0485	0.2420	0.0492
Pyruvate metabolism	0.0485	0.2420	0.0492
Pentose phosphate pathway	0.0492	0.2420	0.0492
Glycerolipid metabolism	0.0492	0.2420	0.0492

Table S11 Effect of roxadustat on the level of energy-carrier molecules in neurons and astrocytes.

Neurons and astrocytes were treated with 20 μM roxadustat for 24 h. DMSO treated cells were used as control. The concentration of energy-carrier molecules was quantified by using LC-MS/MS technique (*n* = 4-7 per group; unpaired two-tailed Student's *t* test).

	**Neurons**			**Astrocytes**		
**compound name**	**mean ± SD (pmol/10^6 cells)**		***p*-value**	**mean ± SD (pmol/10^6 cells)**		***p*-value**
	**control**	**Roxadustat**		**control**	**Roxadustat**	
Adenosine	2.40 ± 1.00	2.45 ± 0.37	0.9243	1.20 ± 0.82	1.09 ± 0.79	0.8300
ADP	723.45 ± 144.52	954.41 ± 98.36	0.0384	521.55 ± 31.39	519.87 ± 26.01	0.9243
ADPR	2.37 ± 0.48	4.18 ± 1.18	0.0292	6.70 ± 4.57	4.52 ± 2.61	0.3617
AMP	91.87 ± 24.34	118.35 ± 16.85	0.1239	66.80 ± 27.84	61.44 ± 31.53	0.7617
ATP	1655.37 ± 302.51	2293.91 ± 362.50	0.0353	2229.86 ± 500.37	2222.99 ± 202.91	0.9776
Cytidine	6.19 ± 0.44	7.28 ± 0.30	0.0065	5.25 ± 0.75	5.05 ± 0.98	0.7044
Cytosine	n.d.	n.d.	/	n.d.	n.d.	/
FAD	7.01 ± 1.13	8.39 ± 0.96	0.1128	8.05 ± 2.90	8.21 ± 2.87	0.9283
GDP	1553.32 ± 390.15	2074.46 ± 409.27	0.1148	680.37 ± 92.30	704.38 ± 81.65	0.6519
GDP-Mannose	88.28 ± 3.61	109.61 ± 18.07	0.0599	63.72 ± 7.45	60.73 ± 6.92	0.4970
GMP	40.77 ± 11.46	49.12 ± 5.83	0.2418	n.d.	n.d.	/
GTP	571.79 ± 62.73	797.12 ± 54.12	0.0016	539.99 ± 60.73	559.69 ± 26.56	0.5160
Guanosine	10.56 ± 1.04	11.95 ± 0.40	0.0469	1.66 ± 0.16	1.92 ± 0.20	0.1479
Hypoxanthine	14.88 ± 2.54	16.93 ± 2.26	0.2737	9.98 ± 3.13	8.98 ± 3.11	0.5944
Inosine	65.05 ± 18.91	70.20 ± 9.25	0.6422	10.58 ± 5.70	8.01 ± 4.77	0.4321

NAAD	118.51 ± 18.12	138.93 ± 7.08	0.0804	77.17 ± 15.01	70.37 ± 10.28	0.4034
NAD	308.44 ± 55.65	379.93 ± 38.80	0.0796	204.93 ± 45.91	185.84 ± 42.30	0.4806
NADH	1016.52 ± 205.59	1409.11 ± 356.88	0.1052	1862.79 ± 401.14	2361.30 ± 220.72	0.0315
NADP	16.76 ± 2.60	34.68 ± 8.72	0.0077	19.67 ± 6.62	18.38 ± 1.98	0.6869
NADPH	1284.44 ± 191.85	1377.43 ± 209.14	0.5366	1441.64 ± 225.12	1249.98 ± 253.28	0.1969
NAMN	59.78 ± 1.01	69.56 ± 5.46	0.0124	55.48 ± 0.37	55.52 ± 0.29	0.8534
NAM	21.25 ± 1.34	30.82 ± 5.64	0.0164	59.32 ± 28.13	57.55 ± 24.87	0.9127
NMN	19.88 ± 2.85	30.49 ± 5.08	0.0108	4.94 ± 0.60	5.06 ± 0.36	0.6998
NR	n.d.	n.d.	/	n.d.	n.d.	/
SAH	2.09 ± 0.32	2.31 ± 0.15	0.2518	n.d.	n.d.	/
SAM	37.47 ± 6.09	44.23 ± 4.25	0.1182	64.01 ± 7.22	66.98 ± 3.72	0.4227
UDP-GlcNAc	777.43 ± 154.04	795.49 ± 112.73	0.8562	2509.38 ± 365.00	2430.62 ± 331.48	0.7103
UDP-Glucose-Galactose	184.57 ± 11.24	212.88 ± 34.61	0.1707	1645.44 ± 366.59	1558.76 ± 154.41	0.6327
Uridine	5.55 ± 0.40	6.57 ± 0.39	0.0105	4.78 ± 0.96	4.61 ± 1.14	0.7792

ADP: Adenosine di-phosphate; ADPR: Adenine diphosphate ribose; AMP: Adenosine mono-phosphate; ATP: Adenosine tri-phosphate; FAD: Flavin adenine dinucleotide; GDP: Guanosine di-phosphate; GMP: Guanosine mono-phosphate; GTP: Guanosine tri-phosphate; NAAD: Nicotinic acid adenine dinucleotide; NAD: Nicotinamide adenine dinucleotide; NADP: Nicotinamide adenine dinucleotide phosphate; NAM: Nicotinamide; NAMN: Nicotinic acid mononucleotide; n.d.: not detectable; NMN: Nicotinamide mononucleotide; NR: NAM riboside; SAH: S-Adenosylhomocystein; SAM: S-Adenosylmethionin; UDP-GlcNAc: Uridine diphosphate-N-acetylglucosamine

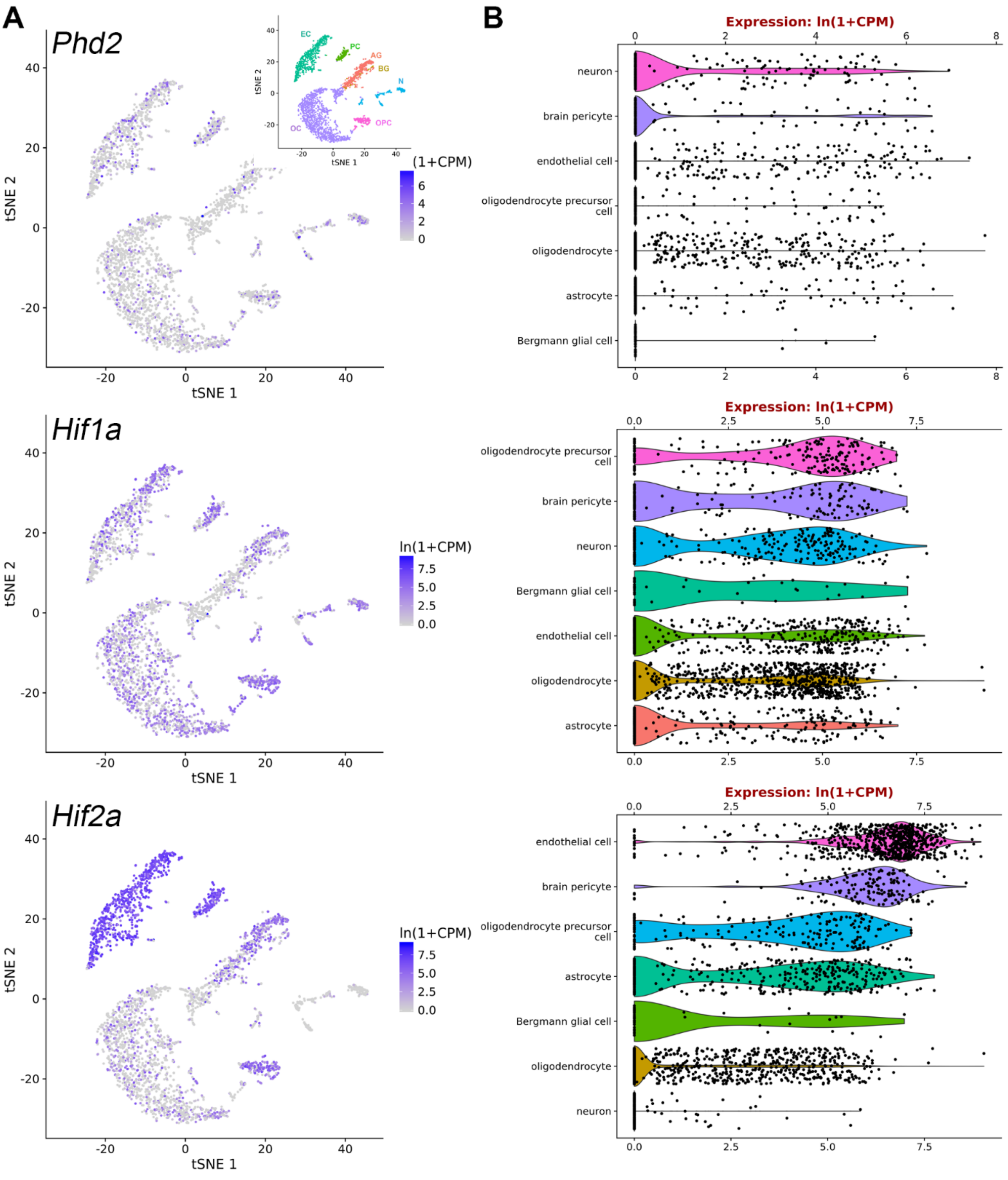

Figure S1 Expression of *Phd2*, *Hif1a* and *Hif2a* in brain-resident cells. **(A)** *t*-SNE plots showing scRNA-seq analysis of *Phd2*, *Hif1a* and *Hif2a* expression in non-myeloid cells of the murine brain (n = 7). Data from the Tabula Muris Consortium [1]. **(B)** Violin plots of log-normalized *Phd2*, *Hif1a* and *Hif2a* counts per million reads (CPM) in non-myeloid brain cells (n = 7). Data from the Tabula Muris Consortium. AG, astroglia; BG, Bergmann glia; EC, endothelial cell; N, neuron; OPC, oligodendrocyte precursor cell; OC, oligodendrocyte; PC, pericyte.

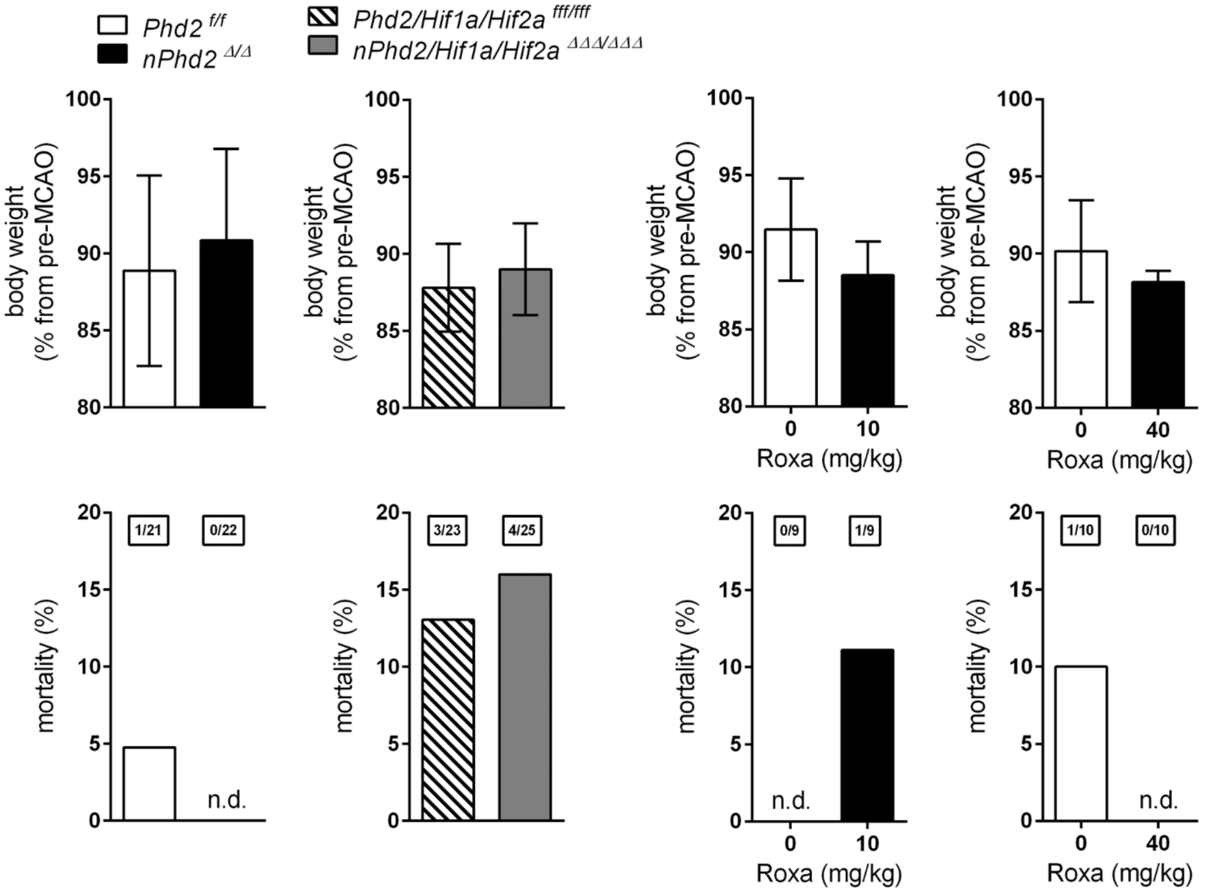

Figure S2 Body weight and mortality after acute ischemic stroke. Mice were subjected to 60 min of MCAO followed by 24 h reperfusion. The body weight was measured before and after ischemia/reperfusion injury, and is shown as relative weight 24 h post-MCAO as compared to values prior to MCAO (mean ± SD; unpaired two-tailed Student's *t* test). The mortality of mice was monitored within 24 h upon onset of reperfusion (mean; Fisher's exact test).

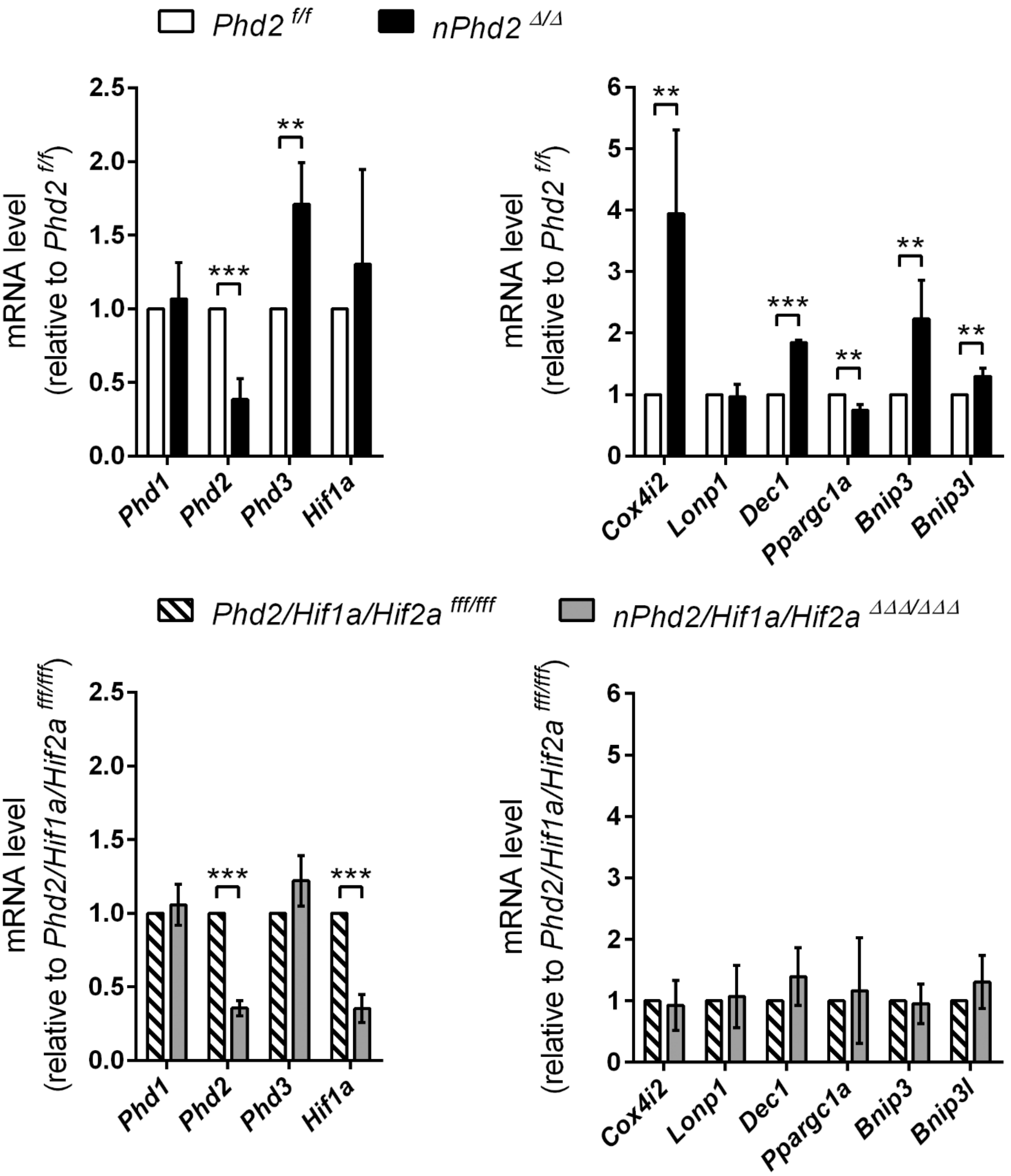

Figure S3 HIF-dependent transcriptional response in cortical neurons derived from transgenic mice lacking members of the neuronal oxygen sensing pathway in neurons. Real-time RT-PCR was used to determine transcript levels of *Phd1-3*, *Hif1a*, and HIF-target genes in primary neurons isolated from brains of neonatal *Phd2* $^{f/f}$, *nPhd2* $^{\Delta/\Delta}$, *Phd2/Hif1a/Hif2a* $^{fff/fff}$ and *nPhd2/Hif1a/Hif2a* $^{\Delta\Delta\Delta/\Delta\Delta\Delta}$ mice. Values are normalized to *Rps12* and expressed as fold change to *Phd2* $^{f/f}$ or *Phd2/Hif1a/Hif2a* $^{fff/fff}$, respectively (mean ± SD; *n* = 4-5 per group; unpaired two-tailed Student's *t* test; ** $p < 0.01$, *** $p < 0.001$).

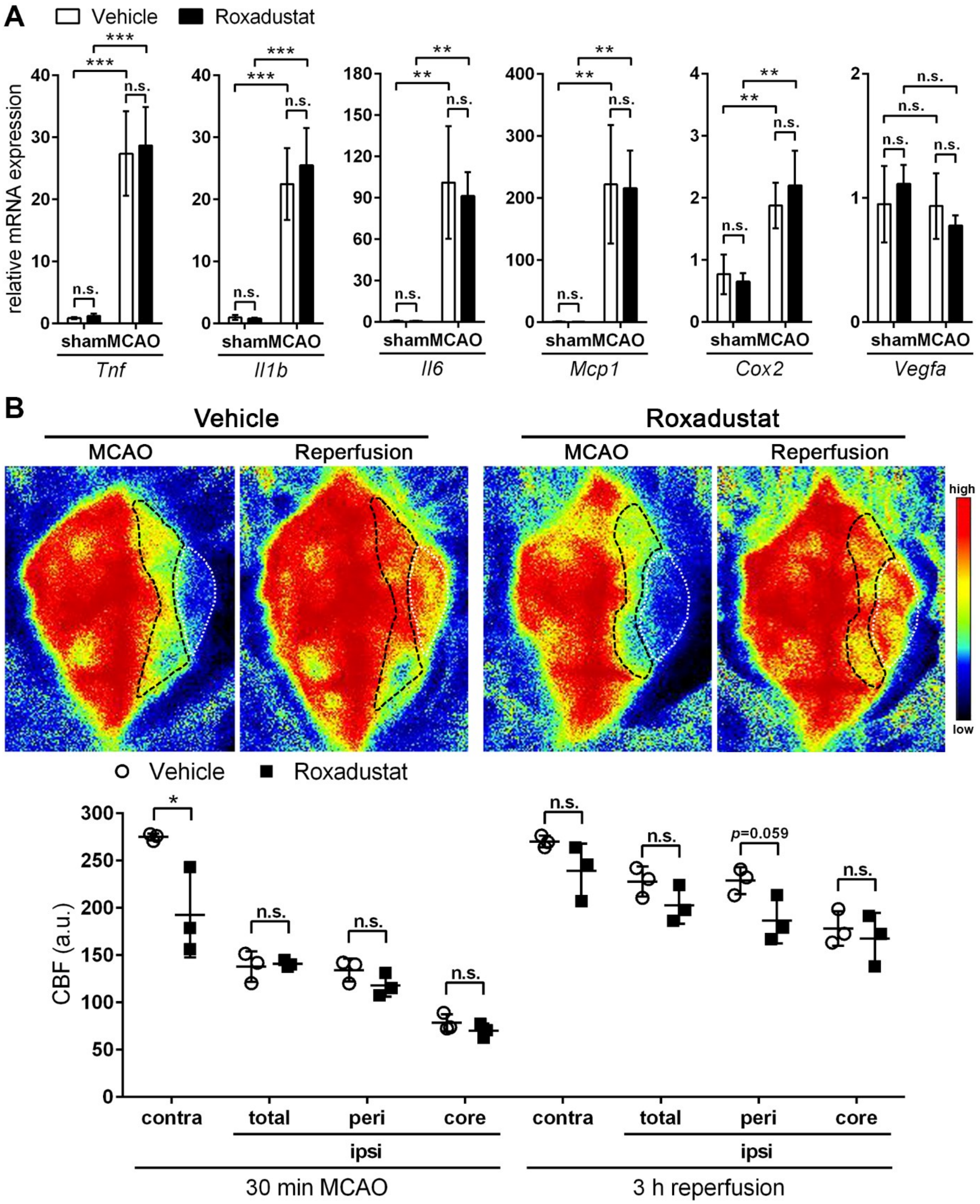

Figure S4 Impact of roxadustat on pro-inflammatory cytokine expression and blood flow reperfusion upon transient cerebral ischemia. Roxadustat was applied intraperitoneally to C57BL/6 mice at a dosage of 40 mg/kg body weight twice daily with 12 h between doses for 4 days. Control mice received equal volumes of vehicle solution. **(A)** Mice were subjected to 60 min of MCAO followed by 24 h reperfusion. Sham-operated mice served as control. Real-time RT-PCR was used to determine transcript levels of pro-inflammatory cytokines. Values are normalized to *Rps12* and expressed as fold change to vehicle treated and sham-operated mice

($n = 3$ per group; Two-way ANOVA with Holm-Sidak's multiple comparisons test; ** $p < 0.01$, *** $p < 0.001$). **(B)** Mice were subjected to 60 min of MCAO followed by 3 h reperfusion. Cortical blood flow (CBF) was measured on the skull of living mice during MCAO and after reperfusion using high-resolution laser speckle contrast imaging (LSCI). Representative LSCI perfusion images of the cortical surface at 30 min MCAO and 3 h upon reperfusion. Color bar code: The colors from blue to red represent the blood flow velocity from lower to higher. CBF is given in arbitrary units (a.u.). ROIs across the ipsilateral brain hemisphere were defined according the following CBF thresholds: infarct core (cortical blood flow < 40% of mean contralateral hemisphere during MCAO), hypoperfused peri-infarct region (cortical blood flow between 40 and 70% of mean contralateral hemisphere during MCAO) ($n = 3$ per group; unpaired two-tailed Student's t test; * $p < 0.05$).

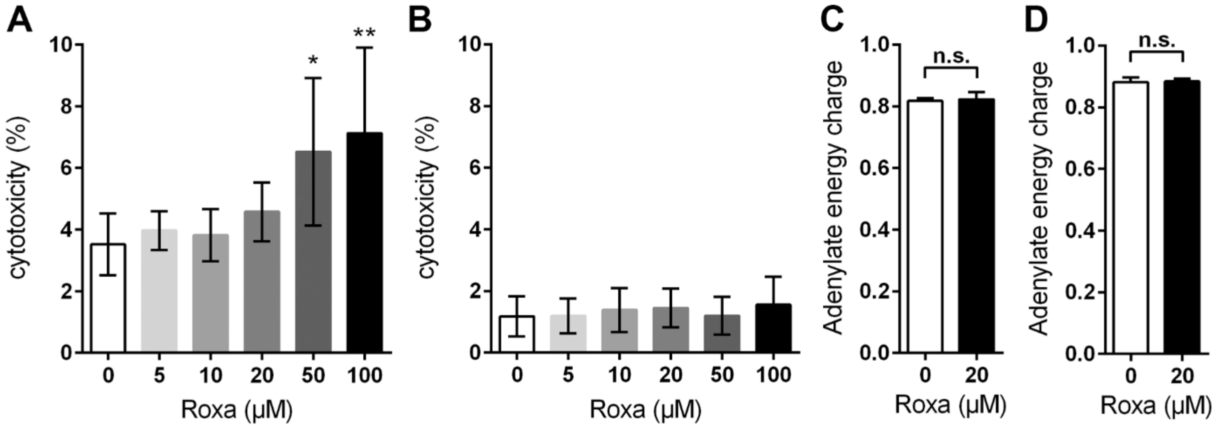

Figure S5 Impact of roxadustat on the viability of neurons and astrocytes. **(A)** Neurons and **(B)** astrocytes were treated with roxadustat at the indicated concentrations for 24 h. DMSO treated cells were used as control. Measurement of lactate dehydrogenase (LDH) activity released from the cytosol of damaged cells into the supernatant was used to determine cytotoxicity. Values are normalized to the total LDH activity released by cell lysis (*n* = 3-6 per group; One-way ANOVA with Holm-Sidak's multiple comparisons test; * $p < 0.05$, ** $p < 0.01$). **(C)** Neurons and **(D)** astrocytes were treated with 20 µM roxadustat for 24 h. DMSO treated cells were used as control. The intracellular concentration of adenosine phosphates was quantified by using LC-MS/MS technique. The adenylate energy charge was calculated as follows: AEC = ($[ATP]_i$ + 0.5 x $[ADP]_i$) / ($[ATP]_i$ + $[ADP]_i$ + $[AMP]_i$) (*n* = 4-7 per group; unpaired two-tailed Student's *t* test).

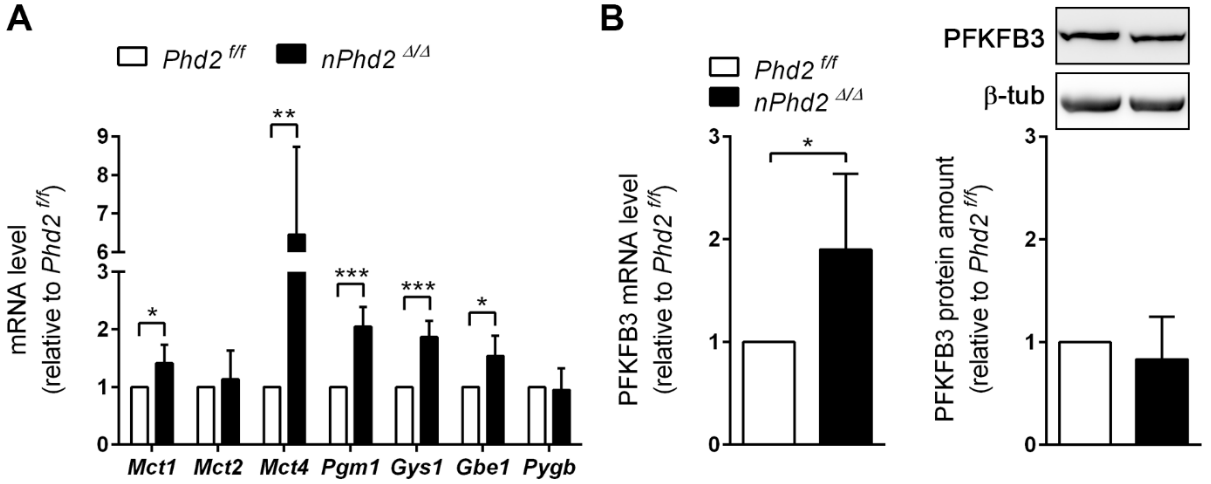

Figure S6 Gene expression in *Phd2* deficient neurons. RNA and proteins were prepared from neurons derived from *Phd2 f/f* and *nPhd2 Δ/Δ* mice. **(A)** Real-time RT-PCR was used to determine transcript levels of monocarboxylate transporter (MCT) isoforms and genes related to glycogen metabolism. **(B)** Real-time RT-PCR and Western blotting were applied to determine transcript and protein levels of PFKFB3. Values are normalized to *Rps12* (mRNA) or β-tubulin (protein) and expressed as fold change to *Phd2 f/f* (n = 3-5 per group; unpaired two-tailed Student's t test; * $p < 0.05$, ** $p < 0.01$, *** $p < 0.001$).

References

1. Tabula Muris C, Overall c, Logistical c, Organ c, processing, Library p *et al.* Single-cell transcriptomics of 20 mouse organs creates a Tabula Muris. *Nature* 2018; 562(7727): 367-372.
